# Characterization of mRNA polyadenylation in the apicomplexa

**DOI:** 10.1371/journal.pone.0203317

**Published:** 2018-08-30

**Authors:** Ashley T. Stevens, Daniel K. Howe, Arthur G. Hunt

**Affiliations:** 1 Department of Plant and Soil Sciences, University of Kentucky, Lexington, Kentucky, United States of America; 2 Department of Veterinary Science, University of Kentucky, Lexington, Kentucky, United States of America; Shanghai Institutes for Biological Sciences, CHINA

## Abstract

Messenger RNA polyadenylation is a universal aspect of gene expression in eukaryotes. In well-established model organisms, this process is mediated by a conserved complex of 15–20 subunits. To better understand this process in apicomplexans, a group of unicellular parasites that causes serious disease in humans and livestock, a computational and high throughput sequencing study of the polyadenylation complex and poly(A) sites in several species was conducted. BLAST-based searches for orthologs of the human polyadenylation complex yielded clear matches to only two—poly(A) polymerase and CPSF73—of the 19 proteins used as queries in this analysis. As the human subunits that recognize the AAUAAA polyadenylation signal (PAS) were not immediately obvious, a computational analysis of sequences adjacent to experimentally-determined apicomplexan poly(A) sites was conducted. The results of this study showed that there exists in apicomplexans an A-rich region that corresponds in position to the AAUAAA PAS. The set of experimentally-determined sites in one species, *Sarcocystis neurona*, was further analyzed to evaluate the extent and significance of alternative poly(A) site choice in this organism. The results showed that almost 80% of *S*. *neurona* genes possess more than one poly(A) site, and that more than 780 sites showed differential usage in the two developmental stages–extracellular merozoites and intracellular schizonts–studied. These sites affected more than 450 genes, and included a disproportionate number of genes that encode membrane transporters and ribosomal proteins. Taken together, these results reveal that apicomplexan species seem to possess a poly(A) signal analogous to AAUAAA even though genes that may encode obvious counterparts of the AAUAAA-recognizing proteins are absent in these organisms. They also indicate that, as is the case in other eukaryotes, alternative polyadenylation is a widespread phenomenon in *S*. *neurona* that has the potential to impact growth and development.

## Introduction

Messenger RNA 3’ end formation, the process by which the 3’-ends of precursor mRNAs are processed and polyadenylated, is an essential step for gene expression in eukaryotes and one at which gene expression may be regulated [1–4]. In mammals, mRNA polyadenylation (or polyadenylation for short) is mediated by a sizeable complex of proteins (more than 15, if one includes isoforms of different subunits of the complex; see [5] for an excellent recent review). This complex includes several biochemically-distinct subcomplexes–Cleavage and Polyadenylation Specificity Factor (or CPSF), Cleavage stimulatory Factor (or CstF), Cleavage Factors I and II (CFIm and CFIIm, respectively)–and other enzymes that bridge these subcomplexes or are recruited by the combined action of one or more of these. These latter include poly(A) polymerase (or PAP, the enzyme that adds the polyadenylated tract to the 3’ end of the cleaved pre-mRNA) and symplekin. These subcomplexes are recruited to the pre-mRNA through interactions with the C-terminal domain of RNA polymerase II and with cis-acting sequence elements within the pre-mRNA. These elements include the mammalian polyadenylation signal AAUAAA (and similar A-rich counterparts in yeast and plants), regions or motifs present 5’ (or upstream) of the polyadenylation signal, motifs situated 3’ (or downstream) of the poly(A) site, and the actual cleavage/polyadenylation site itself. Several individual subunits of the complex bind directly to these motifs. For example, the so-called CstF64 subunit (and a related subunit CstF64 tau) recognizes motifs situated downstream of the poly(A) site [6, 7]. Another subunit, CFIm-25, recognizes the motif UGUA [8, 9] and associates with upstream sequences [6]. Two other subunits, WDR33 and CPSF30, bind to the AAUAAA motif [10, 11].

The relative position at which the precursor mRNA is cleaved and polyadenylated (termed herein as the polyadenylation site, or PAS) determines the ultimate protein-coding potential of the mRNA as well as other functional aspects of the mRNA (such as subcellular location, translatability, and stability). Most eukaryotic pre-mRNAs have more than one potential PAS, and the choice of site often can vary during development or in response to environmental stimuli [3, 4, 12–14]. These variations can affect mRNA stability and function, and help to tune overall transcriptional outputs and mRNA levels. As may be inferred from the plethora of reviews on the subject (but a handful are cited here, in the interest of brevity), alternative polyadenylation (or APA) is widespread in animals and plants, and touches on most aspects of growth and development.

To date, the consensus picture of polyadenylation and its impact on gene expression comes largely from studies conducted in a small number of systems–various animals, yeast, and higher plants. This consensus would seem to be broadly applicable; for example, trypanosomes possesses a similar complement of polyadenylation complex subunits, and these exist in analogous subcomplexes (CPSF, for example) and are important for polyadenylation and organismal survival [15–18]. However, other reports suggest that the process of polyadenylation may accommodate a great deal of variability or flexibility, in terms both of the nature of the *cis* elements that guide polyadenylation and the complex that mediates the process. For example, in studies of poly(A) signals in green algae, motifs related to UGUAA (and not AAUAAA) were found to be most often associated with poly(A) sites [19–22]; in these organisms, there was little indication that an A-rich motif analogous to the mammalian AAUAAA is involved in polyadenylation. In *Giardia lamblia*, variations of the motif GUAA were among the most prominent in regions nearby poly(A) sites; the motif AGUGAA was also a frequent occurrence in this region [23]. In *Trichomonas vaginalis*, the motif UAAA functions as a poly(A) signal [24]. U-rich motifs, typified by UUACUU and UGUUUG, have been proposed to serve as poly(A) signals in *Trypanosoma cruzi* and *Blastocystis hominis*, respectively [19]. Polyadenylation in these latter organisms has additional special features or aspects. In trypanosomes, mRNAs are generated from polycistronic precursors by a combination of trans-splicing (that appends a common leader to mRNAs) and 3’-end polyadenylation; these processes may be coupled, as depletion of at least one poly(A) complex subunit inhibits precursor processing and maturation [17]. In *Blastocystis*, the translation termination codons of many mRNAs are formed by the process of polyadenylation; in these instances, the mRNAs are devoid of 3’-untranslated regions apart from the poly(A) tract itself [25]. These interesting variations of the process raise the possibility of a plethora of mechanisms in other eukaryotes.

The phylum Apicomplexa includes a number of organisms of interest and importance, including pathogens of humans (*Plasmodium* species and *Toxoplasma gondii*) and several species that cause disease in livestock. The process of polyadenylation in these organisms is not well understood. Early reports indicated that, as is seen in plants and yeast, poly(A) sites in *Plasmodium* may occur in clusters in 3’-UTRs that are typically close to motifs similar to AAUAAA [26–28]. More recently, a genome-scale study identified poly(A) sites associated with more than 3400 genes in *P*. *falciparum* [29]; in this study, no analysis of potential poly(A) signals was reported. A subunit of the core poly(A) complex is a target of a novel antiprotozoal compound in *T*. *gondii* [30] and *P*. *falciparum* [31]. Given the latter reports, further study of the process of polyadenylation in these two organisms, and also in other apicomplexans of agricultural importance, is needed. In this report, a bioinformatics and genome-wide sequencing study of mRNA polyadenylation in *Sarcocystis neurona*, *Neospora caninum*, and *T*. *gondii* is presented. The results suggest that the polyadenylation complexes in apicomplexans may differ substantially from those seen in model eukaryotes. They also reveal a probable polyadenylation signal that is similar to those seen in plants and mammals. Finally, they suggest the occurrence of changes in poly(A) site choice during asexual development of *S*. *neurona*.

## Materials and methods

### Bioinformatics

To identify potential apicomplexan polyadenylation complex subunits, TBLASTN was used to search the genomes of *P*. *falciparum*, *T*. *gondii*, *and S*. *neurona*. TBLASTN was chosen to allow for the identification of possible protein-coding regions that have not yet been annotated by the respective communities. The three apicomplexan genomes were downloaded from Plasmodb and Toxodb; unmasked genome sequences were used for this study. The *Arabidopsis* genome was included in this analysis to provide examples of identification of potential subunits in a genome from a phylum apart from metazoans; the genome sequence was downloaded from the TAIR website. The human polyadenylation complex subunits used as queries in this study are listed in Table 1. To present the results, the “e-value” of the best match for each subunit was transformed by taking the -log(10) value for each hit; these transformed values were then plotted.

**Table 1:** Human poly(A) complex subunits used as queries for TBLASTN.

Name	Description	Size	Accession
HsCPSF160	PREDICTED: cleavage and polyadenylation specificity factor subunit 1 isoform X3 [Homo sapiens].	785	XP_006716613
HsCPSF100	cleavage and polyadenylation specificity factor subunit 2 [Homo sapiens].	782	NP_059133
HsCPSF73	cleavage and polyadenylation specificity factor subunit 3 [Homo sapiens].	684	NP_057291
HsC73(2)	integrator complex subunit 11 isoform 2 [Homo sapiens].	600	NP_060341
HsCstF77	cleavage stimulation factor subunit 3 isoform 1 [Homo sapiens].	717	NP_001317
HsCstF50	cleavage stimulation factor subunit 1 [Homo sapiens].	431	NP_001315
HsWDR33	PREDICTED: pre-mRNA 3' end processing protein WDR33 isoform X2 [Homo sapiens].	1133	XP_005263754
HsCFIm25	cleavage and polyadenylation specificity factor subunit 5 [Homo sapiens].	227	NP_008937
HsClp1	polyribonucleotide 5'-hydroxyl-kinase Clp1 isoform 2 [Homo sapiens].	361	NP_001136069
HsPcf11	PCF11p homolog [Homo sapiens].	725	AAC03107
HsPABN1	BCL2L2-PABPN1 protein [Homo sapiens].	333	NP_001186793
HsSymplekin	PREDICTED: symplekin isoform X2 [Homo sapiens].	936	XP_011525657
HsCFIm68	Human pre-mRNA cleavage factor I 68 kDa subunit [Homo sapiens].	551	CAA47752
HsCPSF30	cleavage and polyadenylation specificity factor subunit 4 isoform 2 [Homo sapiens].	244	NP_001075028
HsCstF64	cleavage stimulation factor subunit 2 isoform 1 [Homo sapiens].	597	NP_001293135 XP_011529175
HsPAPa	poly(A) polymerase alpha, isoform CRA_f [Homo sapiens].	744	EAW81649
HsCstF64tau	Cleavage stimulation factor, 3' pre-RNA, subunit 2, 64kDa, tau variant [Homo sapiens].	616	AAH28239
HsPAPg	poly(A) polymerase gamma, isoform CRA_f [Homo sapiens].	736	EAX00034
HsFip1	FIP1 like 1 (S. cerevisiae), isoform CRA_e [Homo sapiens].	565	EAX05449

Virtual northern blots were generated using a *S*. *neurona* RNASeq dataset (accession SRR1784218); these reads were generated from RNA isolated from *S*. *neurona* SO SN1 merozoites [32] and were used without modification. Mapping was performed using the Large Gap Read Mapping tool in the CLC Genomics Workbench package with the following parameters: References = the genomic regions surrounding the respective genes, Maximum number of hits for a segment = 10, Maximum distance from seed = 50,000, Multi match mode = ignore, Mismatch cost = 2, Insertion cost = 3, Deletion cost = 3, Similarity = 0.9, Length fraction = 0.5, Override default distances = No, Create Report = Yes, Create non mapped list = No.

### Cell culturing and isolation of DNA and RNA

*Sarcocystis neurona* (strain SN3.E1), *T*. *gondii* (strain RH), and *N*. *caninum* (strain NC-1) were propagated by serial passage in monolayers of BT cells, as described [33]. Upon lysis of the infected monolayers, extracellular zoites were harvested by passing through 23 and 25-gauge needles and filter-purified to remove host cell debris. Zoites were pelleted and stored at -80°C until used for DNA or RNA isolation.

For analysis of intracellular parasites, freshly egressed merozoites were inoculated onto BT cell monolayers and allowed to invade for 1 hr. Extracellular zoites were removed from the cultures by washing 2X with PBS and then incubated for 48 hrs to allow schizont development. The infected monolayer was harvested from the flask using a cell scraper, pelleted, and stored at– 80°C until used.

For isolation of genomic DNA from *S*. *neurona*, harvested merozoites were incubated overnight in 10 mM Tris-HCL (pH 8.0), 100 mM EDTA, 1% sarkosyl, 2 mg/ml proteinase K, followed by extraction with phenol-chloroform and precipitation with 2 volumes 95% ethanol. The resulting nucleic acid pellet was resuspended in TE containing RNase A and stored at 4°C.

RNA was isolated from parasites or infected host cells by adding 1mL Trizol to cell pellets, incubating for 5 minutes at room temperature, then adding 200 μL chloroform and incubating for 3 minutes at room temperature. Samples were centrifuged at 12,000 RPM for 15 minutes, and the top layer was transferred to a fresh tube. 500 **μL** of isopropanol was added and mixed, and allowed to sit overnight at -20°C. The tubes were then spun at 12,000 RMP for 10 minutes and the pellet was washed with 1 mL 75% EtOH. The pellet was air dried for 5–10 minutes and resuspended in RNase free water.

### Poly(A) site profiling

Libraries for genome-wide poly(A) site profiling was performed following the procedures described in Ma *et al*. [34] and Pati *et al*. [35], using the oligonucleotide primers described in S1 Table. So-called poly(A) tag (PAT-Seq) libraries were sequenced on an Illumina MiSeq instrument; the sequencing data are available under Bioproject ID PRJNA436572. Sequencing reads were trimmed, demultiplexed, and mapped to the respective genomes using CLC Genomics Workbench (latest version used was 10.1); the results of these analyses are summarized in S1 File. Subsequent analyses were performed following the pipelines described in Bell *et al*. [36] and de Lorenzo *et al*. [37]. It is important to note that, prior to defining poly(A) sites and quantifying poly(A) site usage, mapped reads were reduced to single nucleotide coordinates that correspond to the mRNA-poly(A) junction defined by the reads. To perform the nucleotide composition study, custom genome annotations for *T*. *gondii*, *S*. *neurona*, and *N*. *caninum* were produced by extending annotated genes by some 500, 500, and 2000 bp, respectively, and then extracting the respective 3’-UTRs; poly(A) sites that mapped to the resulting extended 3’-UTRs were used for this study. (The lengths of these extensions were arrived at empirically, by systematically mapping reads to annotations with differing lengths of extensions, and choosing the lengths that yielded the greatest number of mapped reads.) A separate revised annotation of the *S*. *neurona* genome was generated using a list of poly(A) site clusters (PACs) produced by grouping individual sites that lie within 24 nts of each other (this parameter is the same as that used in refs. 34 and 35); in this annotation, PACs that mapped to annotated 3’-UTRs were identified and, where appropriate, used to extend to annotations. The extended genes were used for the gene expression analyses.

To evaluate global shifts in mRNA lengths, a weighted normalized mRNA length for genes impacted by APA was calculated for each condition (merozoites and schizonts). For this, the fractional usage of each site was multiplied by the fractional length of the corresponding mRNA, with 100% usage at a single site being set as 1.0, and the length of the longest mRNA being set as 1.0. Fractional mRNA lengths were calculated using the corrected *S*. *neurona* annotation described in the preceding paragraph. The fractional usages of all PACs for each gene were then summed, and the differences between these values in merozoites and schizonts calculated; positive values denote increased usage of distal poly(A) sites in merozoites. The results were binned into increments of 0.05, the fraction of all affected genes whose values fell within the different increments were tabulated and the results plotted as shown. More details may be found in S2 File.

### Gene expression analysis

Gene expression was estimated by mapping individual PAT-Seq reads to the revised *S*. *neurona* genome annotation indicated in the preceding subsection using Bedtools as described elsewhere [37, 38]; the utility of 3’-end tags for quantifying gene expression is discussed in Lohman et al. [39]. The resulting mappings of raw reads were ported into CLC Genomics Workbench and the gene expression tools in the package used to quantify relative expression. The process and results are explained in detail in S3 File.

## Results

### A bioinformatics study of the apicomplexan polyadenylation complex

While the polyadenylation complexes of animals and yeast have been extensively characterized, less is known about analogous complexes in most other eukaryotes. However, a conclusion drawn by the authors of a study of the genome of *G*. *lamblia* is provocative [40]; these authors noted that the genome of this organism seems to possess genes encoding only three of the subunits (CPSF73, CPSF30, and PAP) seen in the mammalian complex.

**Fig 1:**
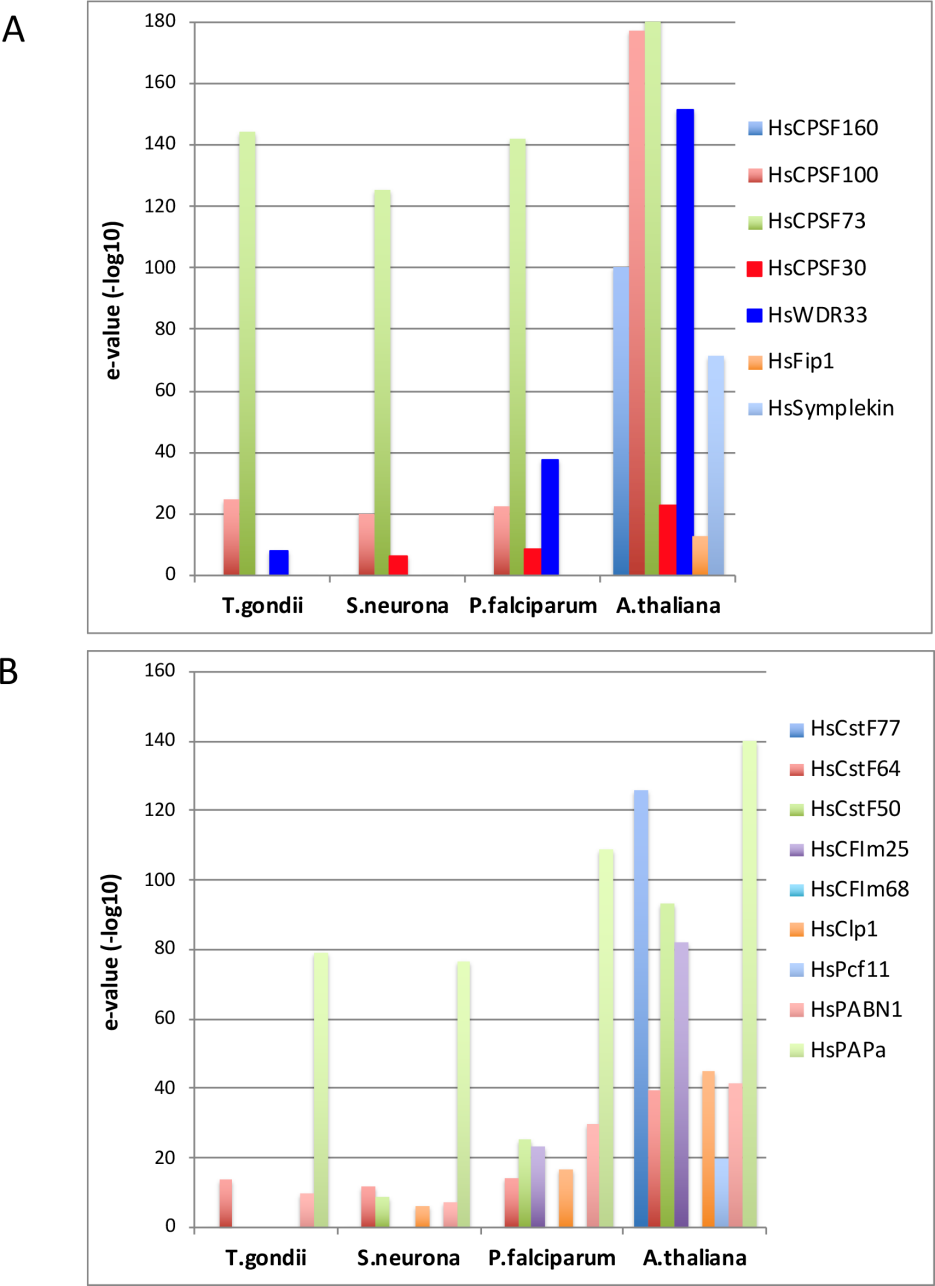
TBLASTN analysis of apicomplexan poly(A) complex subunits. Results of a TBLASTN search was conducted using the collection of human polyadenylation complex subunits as queries against three apicomplexans genomes. *A*. *thaliana* was included to illustrate results obtained when orthologs exist. The y-axis on this plot is the negative log(10) of the e-value of the strongest match obtained in the BLAST search. The plot on the left shows the results obtained with CPSF subunits, and that on the right those obtained with other subunits. In these plots, values greater than 20 may be taken to denote possible orthologs. CPSF subunits are shown in panel A, and other subunits in panel B.

With this curious result in mind, a TBLASTN analysis of three apicomplexan genomes–*P*. *falciparum*, *S*. *neurona*, and *T*. *gondii*–was conducted, using the suite of human polyadenylation complex subunits as queries. For comparative purposes, the *Arabidopsis* genome was also searched. TBLASTN was chosen for this study to allow for the identification of possible orthologs in situations in which the community annotations have yet to identify the respective proteins. The only clear matches in any of the apicomplexan genomes were to CPSF73 and PAP (Fig 1A and 1B; panels A and B in S1 Fig). There were also suggestive, although less than definitive, matches for WDR33, PABN1, and CFIm25. The alignments of the closest apicomplexan matches to the latter three human proteins are shown in S1 Fig (panels C-E). To provide context, the sequences of respective orthologs from *Arabidopsis*, rice, and *Physcomitrella patens* (moss) were included in these alignments. As can be seen, the bulk of the sequence similarities between the apicomplexan and human proteins involve commonly-occurring sequence motifs (the WDR repeat in the case of WDR33, the RRM motif in the case of PABN1, and the NUDIX motif in the case of CFIm25).

Whereas clear orthologs of one of the two mammalian subunits that recognize the AAUAAA motif (WDR33) are not apparent in the three apicomplexan genomes studied here (panel C of S1 Fig), the situation is less clear for possible counterparts of the other protein, CPSF30 (or CPSF4) that also contacts AAUAAA. There is a protein in the three apicomplexan species that shows limited similarity to the human CPSF30 (panel F of S1 Fig). This similarity reflects the presence, in the apicomplexan and human proteins, of an array of three CCCH zinc finger motifs (the respective human domain is boxed in red in panel F of S1 Fig). The apicomplexan proteins lack two of the CCCH motifs seen in the human protein (as well as its yeast counterpart; these motifs are highlighted by the green boxes in panel F of S1 Fig). However, when selected plant CPSF30 proteins are included in the alignments, a more pronounced similarity is seen. This reflects several novel features of the plant proteins–the presence of the three (and not five) CCH motifs and also of a domain that bears resemblance to the so-called YTH domain (highlighted by the blue boxes in panel F of S1 Fig). The latter domain is associated with proteins that bind to RNAs in which particular adenosine bases have been modified by methylation at the N6 position. The CPSF30-YTH arrangement is a feature seen in all known examples of higher plant CPSF30 proteins [41]. The three apicomplexan proteins show significant similarity in the CCCH motifs, and also in the YTH domain (panel F of S1 Fig). This arrangement is confirmed by mapping of RNA-Seq reads *S*. *neurona* (panel G of S1 Fig). These results show that the parts of the putative *S*. *neurona* CPSF30 gene that encode the zinc finger arrays and the YTH domains may be connected by individual rRNA-Seq reads, providing support for the hypothesis that, as is the case in plants, these two domains are present in a single polypeptide.

**Fig 2:**
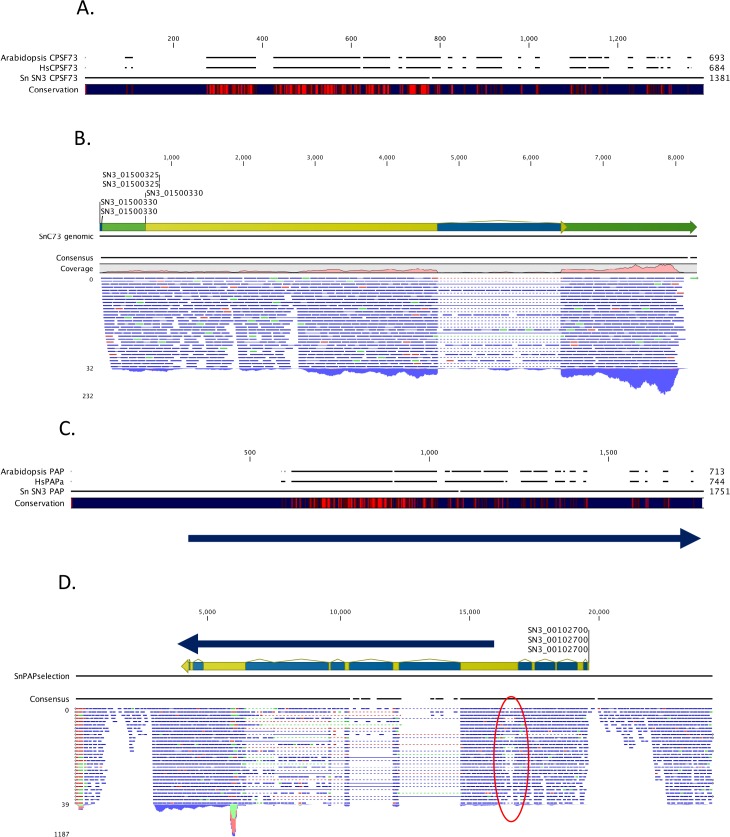
Expression of the S. neurona CPSF73 and PAP genes. A. Alignment of the S. neurona CPSF73 amino acid sequence (SN3_01500330_CPSF73) with the CPSF73 sequences from Arabidopsis (A. thaliana CPSF73) and humans (HsCPSF73). The extent pf conservation among the three sequences is depicted on the “Conservation” line, with red denoting high sequence conservation and dark blue low conservation. Analyses and depictions were done using CLC Genomics Workbench. B. Characterization of mRNAs encoding the *S*. *neurona* CPSF73 protein. RNA-Seq reads were mapped to the region of the *S*. *neurona* genome that includes the predicted CPSF73 gene using CLC Genomics Workbench. Mapping results were presented as genome browser tracks. Blue tags denote authentic paired-end reads, whereas green and red tags denote broken reads. C. Alignment of the predicted *S*. *neurona* PAP amino acid sequence (Sn SN3 PAP) with the PAP sequences from *Arabidopsis* (A. thaliana PAP) and humans (HsPAP), and with the corrected *S*. *neurona* polypeptide discussed in the text (Sn SN3 PAP revised). The extent pf conservation among the four sequences is depicted on the “Conservation” line, with red denoting high sequence conservation and dark blue low conservation. Analyses and depictions were done using CLC Genomics Workbench. The location of possible PAP-encoding sequences whose annotation is supported by RNA-Seq reads is shown by the arrows below the alignment. D. Characterization of mRNAs encoded by the region that includes the predicted *S*. *neurona* PAP gene. RNA-Seq reads were mapped to the region of the *S*. *neurona* genome that includes the predicted PAP gene using CLC Genomics Workbench. Mapping results were presented as genome browser tracks. A possible discontinuity in transcription in this region is highlighted with a red oval. The location of possible PAP-encoding sequences whose annotation is supported by RNA-Seq reads is shown by the arrows above the annotation.

There are two poly(A) complex subunits that can be identified in the apicomplexan genomes, CPSF73 and PAP. In animals, plants, and yeast, CPSF73 is highly conserved in both sequence and size. The apicomplexan orthologs are predicted to be considerably larger, with central region that is similar to the human CPSF73 and a N- and C- terminal domains that is absent in other eukaryotic counterparts (Fig 2A, panel A in S1 Fig). To confirm that the annotations of the *S*. *neurona* CPSF73 gene are accurate, *S*. *neurona* RNA-Seq reads were mapped to the region of the *S*. *neurona* genome that includes the predicted CPSF73 gene. The results confirm the accuracy of the annotation, supporting the existence of a single ca. 8000 nt mRNA (Fig 2B). No indications of splicing or other modifications that might interrupt the protein-coding region were apparent, again supporting the conclusion that the *S*. *neurona* CPSF73 protein is much larger than its other eukaryotic counterparts.

Apicomplexan genes encoding poly(A) polymerase (PAP) are also apparent in the BLAST analysis shown in Fig 1. As is the case with CPSF73, the apicomplexans PAP is predicted to be much larger than its plant and human counterparts (Fig 2C, panel B in S1 Fig). To confirm the accuracy of this annotation, *S*. *neurona* RNA-Seq reads were mapped to the region of the *S*. *neurona* genome that includes the predicted PAP gene (Fig 2D). In contrast to what is seen with the *S*. *neurona* CPSF73 gene, the transcriptomics analysis of the *S*. *neurona* PAP gene annotation suggest that the annotation may be inaccurate, but that the *S*. *neurona* PAP is indeed much larger than its other eukaryotic counterparts. Specifically, the annotation corresponding to the 5’ end of the putative PAP-encoding mRNA is not well supported by the RNA-Seq reads, in that there is little support for the splicing pattern in this annotation. RNA-Seq support for the structure of the remaining portion of the PAP-encoding gene is stronger; this portion includes all of the sequences that bear similarity to other PAPs (Sn SN3 PAP revised in Fig 2C). It is difficult to reconcile the two portions of the predicted gene with a single polypeptide, as the unspliced 5’ portion of the predicted mRNA would result in a very long 5’-untranslated region with numerous upstream AUG codons preceding any possible start codon for the PAP coding region. A possible discontinuity in the RNA-Seq reads (highlighted with the red oval in Fig 2D) raises the possibility that this locus may give rise to two mRNAs; the sequences whose processing pattern is supported by RNA-Seq reads (see the arrow over the annotation in Fig 2D) would encode a polypeptide with all of the PAP-related sequences. However, even given this correction of the *S*. *neurona* PAP annotation, the predicted protein would still be considerably larger than the *Arabidopsis* and human orthologs. Therefore, as is the case with CPSF73, the *S*. *neurona* PAP would seem to be a larger protein than its eukaryotic counterparts.

### The apicomplexan species possess an A-rich polyadenylation signal

In mammals, two subunits of the poly(A) complex–WDR33 and CPSF30 –bind to the AAUAAA motif in the pre- mRNA [10, 11, 42]. As indicated above (Fig 1), no clear orthologs of WDR33 were identified in the three apicomplexan genomes. This observation raises questions regarding the nature of the poly(A) signal in apicomplexans. To examine this, a global study of poly(A) sites in three apicomplexans—*S*. *neurona*, *N*. *caninum*, *and T*. *gondii*—was conducted, with the purpose of characterizing sequences surrounding poly(A) sites in these organisms. For this, RNA was isolated from the three organisms at two distinct stages of growth—from extracellular parasites and from parasite-infected host cells. The isolated RNA was used to prepare so-called poly(A) tag libraries and these libraries were sequenced on the Illumina platform. The sequence data were analyzed using the methods described previously [36, 37] so as to identify polyadenylation sites. In these analyses, the data from extracellular and intracellular parasites were pooled to provide an overall view of possible poly(A) signals. The analysis focused on sites that mapped to annotated 3’-UTRs, or to reconstructed 3’-UTRs in the case of annotated genes lacking this feature (see Methods). Subsequently, the nucleotide composition surrounding these sites was determined.

**Fig 3:**
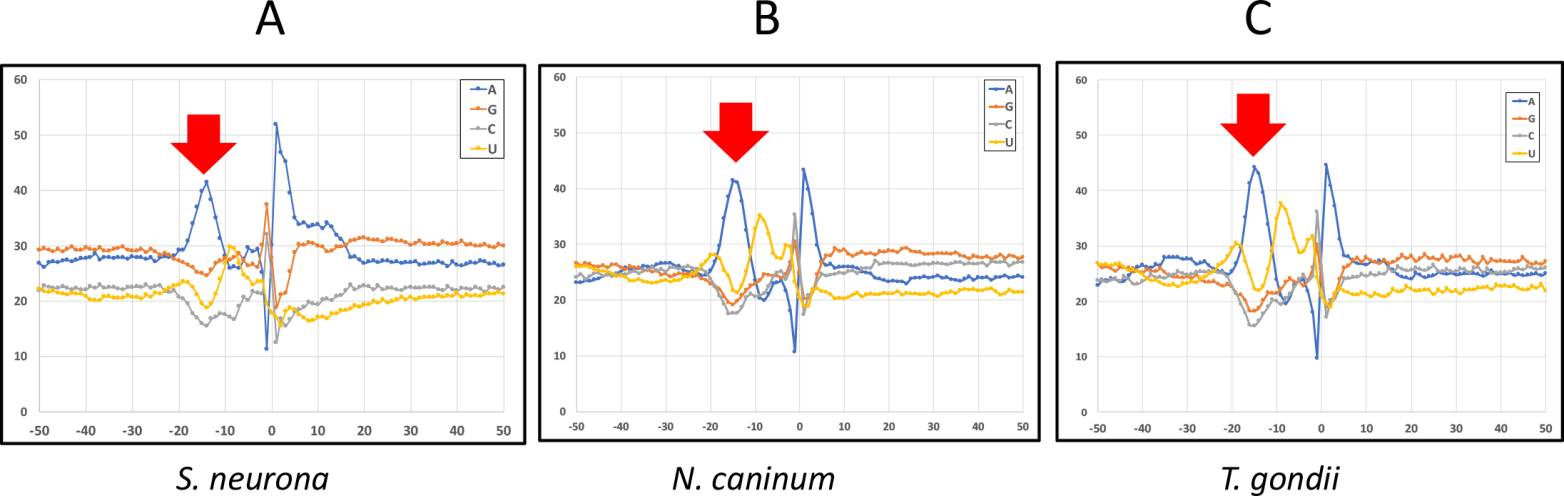
Nucleotide composition surrounding poly(A) sites in three apicomplexan parasites. Poly(A) sites were identified using high throughput PAT-Seq data and the nucleotide compositions determined as described [36, 43, 44]. Datapoints represent the relative fractional composition of each nucleotide at each position extending from 50 nts upstream to 50 nts downstream of the experimentally-determined or mock sites. The organism under study is indicated beneath each plot. Red arrows denote the prominent A-rich region situated upstream from the poly(A) site.

For these three organisms, a distinctive and common nucleotide base composition surrounding poly(A) sites was observed (Fig 3). There was a decided region of A-richness around 20 nts 5’ (upstream) from the poly(A) site and a possible (G,C)A dinucleotide at the processing site itself. A motif analysis failed to identify highly-abundant motifs (such as AAUAAA) in this region, or elsewhere in the vicinity of these collections of poly(A) sites (not shown).

An additional feature, a bias for A immediately following the poly(A) site, was also seen in all three organisms (Fig 3). This latter feature raises the possibility of internal priming by the oligo-dT primer used for the library preparation. However, two facts argue against this. The first is that tracts of 6 or more A were masked in the mapping process, thus eliminating from the study sequences possibly derived from internal priming at modest tracts of A. In addition, similar computational analyses of the regions surrounding oligo-A tracts in 3’-UTRs failed to show the distinctive A-rich upstream region or other base composition trends in (S2 Fig). This argues against extensive internal priming by RT, since this would be expected to yield results in Fig 3 similar to those shown in S2 Fig.

### Alternative polyadenylation in *S*. *neurona*

Intracellular development of *T*. *gondii* and *N*. *caninum* occurs by endodyogeny, a relatively rapid process (6–8 hours) that produces two daughter zoites from a mother cell. In contrast, *S*. *neurona* propagates by endopolygeny, which progresses over approximately 72 hours and produces 64 daughter merozoites from a single mother cell schizont. This lengthy developmental process permits preparation of RNA samples from parasites that are distinctly extracellular (invasive form) or intracellular (propagative form). Accordingly, the poly(A) site profiles of *S*. *neurona* in the extracellular merozoite (M) and intracellular schizont (S) stages were determined and compared to identify poly(A) sites and associated genes whose usage varies significantly during asexual development. For this analysis, individual poly(A) sites situated near other sites were grouped into clusters as described elsewhere [36]. This process yielded 26,560 poly(A) site clusters (or PACs; S4 File). Of these, 24348 could be associated with at least one annotated gene; 23831 could be linked with one annotation, while 516 were associated with two or more adjacent annotations (S4 File). These PACS defined poly(A) sites in 4594 genes, or about 65% of all genes in the current *S*. *neurona* genome annotation.

At least two PACs were observed in 79% of all *S*. *neurona* genes (S3 Fig), revealing a possibility of extensive APA. Accordingly, an analysis of possible APA in the merozoites and schizonts in *S*. *neurona* was conducted. The results showed that 306 PACs exhibited highly-significant differences in usage in the two stages (S5 File). These 306 sites were associated with 204 gene annotations in the *S*. *neurona* genome. The number of sites showing less-significant but still detectable APA was 783; these sites affect 454 genes, which is more than 6% of annotated *S*. *neurona* genes [32]. This analysis suggests a significant extent of APA in *S*. *neurona* during asexual development. Interestingly, the set of 454 genes impacted by APA in *S*. *neurona* includes a significant number that encode membrane transporters and ribosomal proteins (Table 2, S5 File).

**Table 2:** Genes encoding transporters and ribosomal proteins that are affected by APA.

Membrane transporters	
Gene ID	description
SN3_00401550	slc30a2 protein
SN3_00601110	sugar transporter st3
SN3_00700860	atp synthase f1 gamma subunit
SN3_00701105	sarco endoplasmic reticulum ca2+-atpase
SN3_00800440	vacuolar atp synthase subunit
SN3_01000240	sulfate transporter
SN3_01300355	cytochrome c oxidase subunit
SN3_01500760	vacuolar proton translocating atpase subunit a
SN3_02300385	atpase synthase subunit alpha
SN3_02400210	hypothetical protein TGVAND_251470
SN3_02500355	abc transporter transmembrane region domain-containing protein
SN3_03000190	h+-translocating inorganic pyrophosphatase
SN3_03100085	vacuolar atp synthase 16 kda proteolipid
SN3_03300085	protein translocation sec61 gamma subunit
Ribosomal proteins	
Gene ID	description
SN3_00100410	40s ribosomal protein
SN3_00103265	ribosomal protein rpl3
SN3_00200730	40s ribosomal protein s18
SN3_00201205	ribosomal protein rpl15
SN3_00201940	ribosomal protein rpl23a
SN3_00301840	ribosomal protein rps4
SN3_00600110	ribosomal protein rpl5
SN3_00600200	ribosomal protein rpl39
SN3_00600255	ribosomal protein rpl26
SN3_00700730	ribosomal protein rpl35a
SN3_00700770	40s ribosomal protein s20
SN3_00701265	ribosomal protein
SN3_00800655	40s ribosomal protein s27
SN3_00801145	mitochondrial large subunit ribosomal
SN3_00900035	ribosomal protein rpl13a
SN3_01500720	40s ribosomal protein
SN3_01600085	ribosomal protein rps21
SN3_01900030	ribosomal-ubiquitin protein rps27a
SN3_02200105	60s acidic ribosomal protein p0
SN3_02500215	ribosomal protein rpl37a
SN3_02800410	40s ribosomal protein s5
SN3_03000045	60s ribosomal protein l23
SN3_03300035	40s ribosomal protein s16
SN3_03300285	ribosomal protein rpl4
SN3_03700215	40s ribosomal protein s23

**Fig 4:**
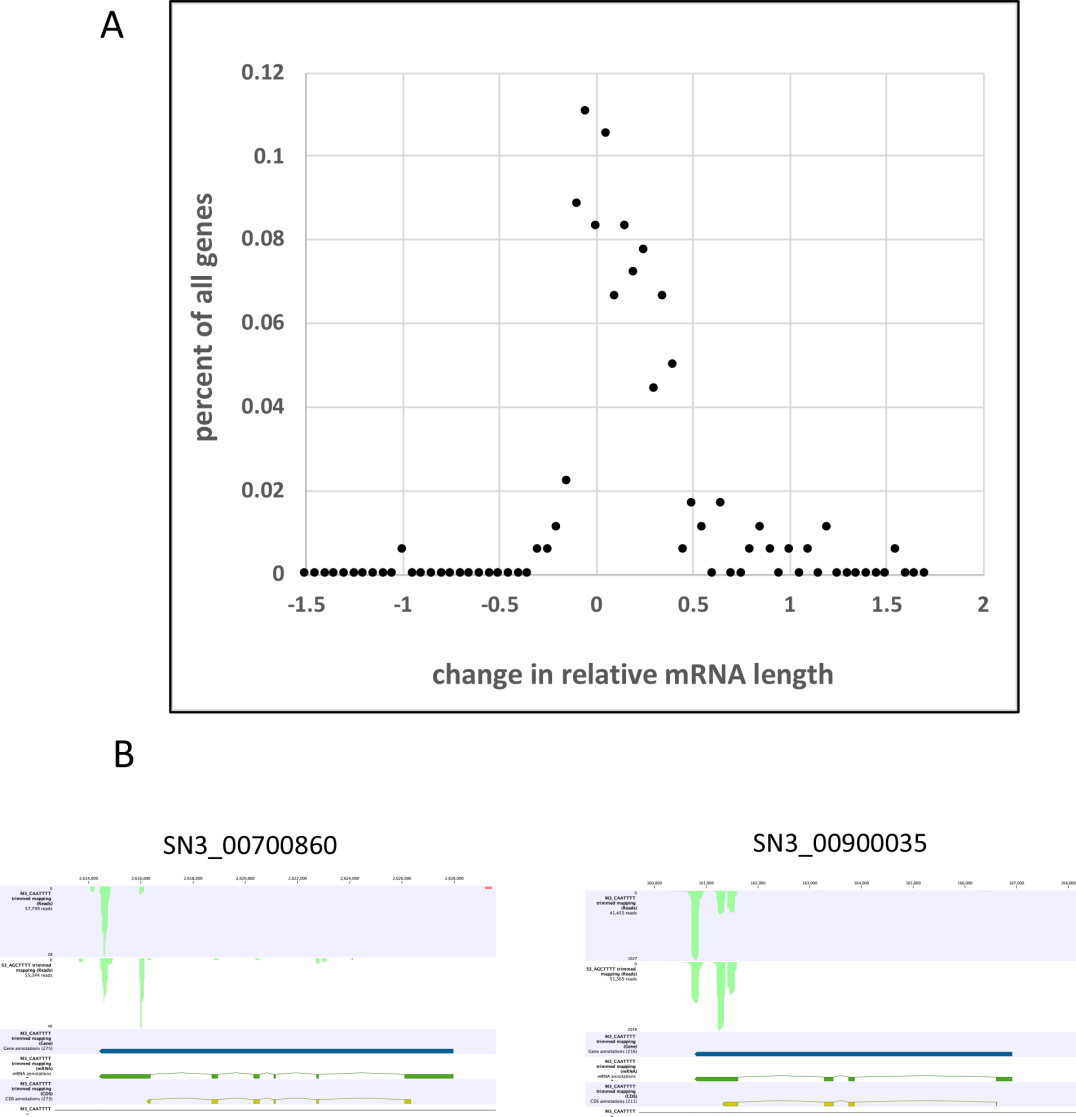
Changes in mRNA lengths for genes affected by APA. A. Analysis of poly(A) site shifts for *S*. *neurona* gene that possess poly(A) sites whose usage changes significantly during development. For each gene, a weighted and normalized mRNA length was calculated as described in Methods, and the differences in this value between the merozoite and schizont stages calculated. The values were binned into discreet groups and the numbers of genes with changes falling into each bin plotted as shown. Positive values denote genes whose mRNA lengths are longer in merozoites than in schizonts. B. Browser tracks showing typical APA patterns for mRNAs encoding a membrane transporter (SN3_00700860) and a ribosomal protein (SN3_00900035). In both cases, the 5’-3’ orientations of these genes are right to left.

One important mechanism by which APA contributes to the regulation of gene expression in animals is via the alteration of the lengths mRNAs, usually involving differential use of sites situated within 3’-UTRs. To test whether this mechanism may apply to apicomplexans, an analysis of the lengths of mRNAs derived from genes impacted by APA in *S*. *neurona* was conducted. For this, a normalized aggregate mRNA length for each gene was calculated; this metric was arrived at by determining the contributions that each mRNA isoform makes to the total numbers of species encoded by the respective gene, and then calculating a normalized aggregate length with the longest isoform being set as 1. When the normalized aggregate lengths for the genes listed in S5 File were compared in the M and S stages, the majority of these genes showed a trend towards longer mRNAs in the M stage (Fig 4A). Instances wherein dramatic or complete switching between two isoforms were rare; rather, the changes reflected quantitative shifts in poly(A) site usage (examples are shown in Fig 4B), usually resulting in changes in the lengths of the 3’-UTR.

### Developmental changes in gene expression in *S*. *neurona*

**Fig 5:**
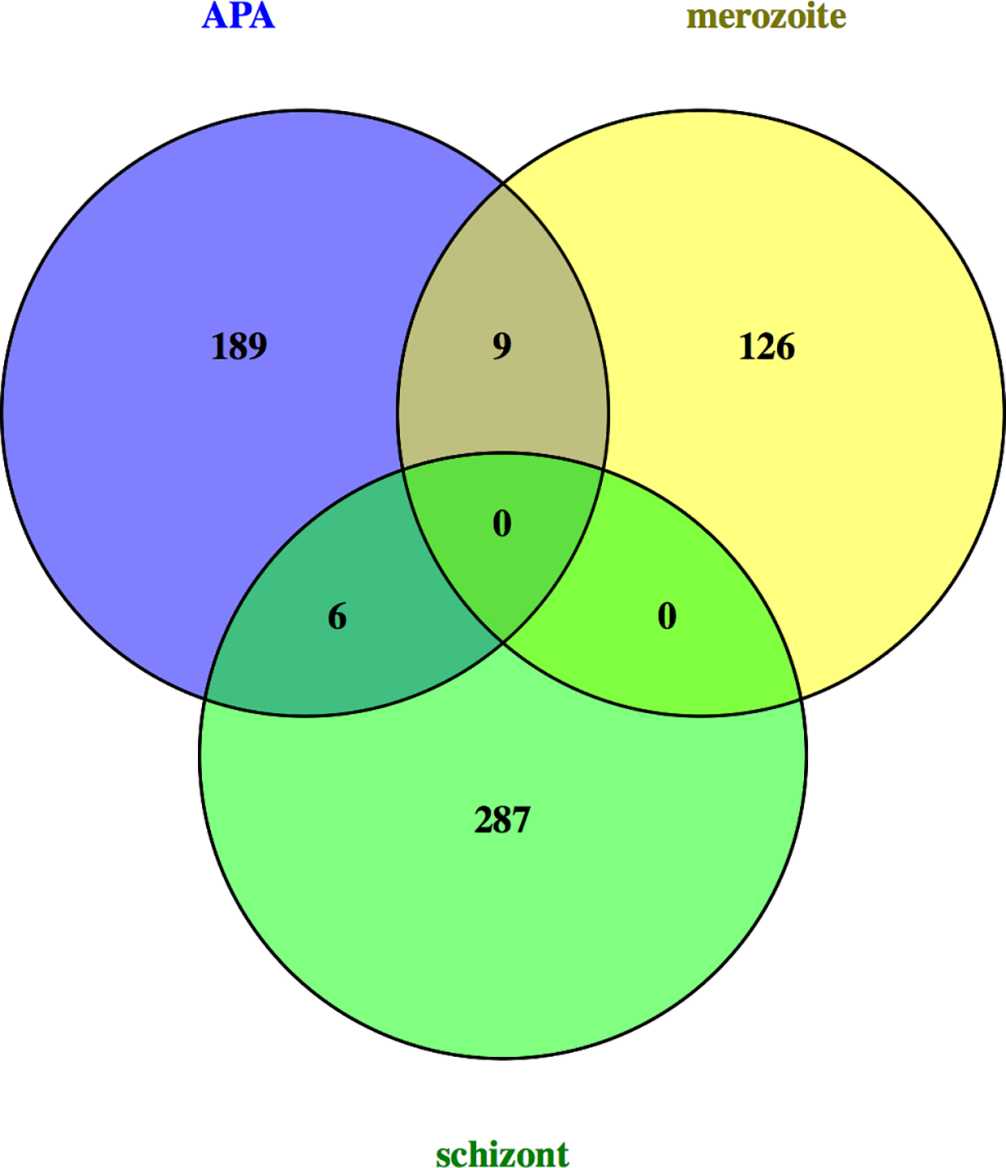
Comparison of genes affected by APA and developmentally-regulated expression. Venn diagram showing the minimal overlap between genes affected by APA, those up-regulated in merozoites, and those up-regulated in schizonts. Gene lists are from S3 and S5 Files.

The poly(A) tag data can provide an assessment of overall gene expression levels. Accordingly, changes in gene expression between the two stages of *S*. *neurona* were determined by quantifying and analyzing the total numbers of poly(A) tag reads that map to *S*. *neurona* genes. This analysis yielded a set of 576 genes whose expression differed significantly in merozoites or schizonts (S3 File). Included in this list were genes that are expected to reflect the different biological needs of the intracellular propagating stage relative to the extracellular invasive stage of *S*. *neurona*. Thus, genes whose expression was at least 5-fold higher in merozoites than schizonts included those that encode proteins associated with gene regulation (transcription factors, protein kinases, and second messenger binding or production; S3 File). Metabolic pathways involving phosphoinositide and sterol metabolism were also disproportionately up-regulated in the extracellular stage. Genes whose expression was at least 5-fold higher in the intracellular stage included those whose products are involved in DNA replication, glycolysis, gluconeogenesis, fermentation, and sphingolipid biosynthesis (S3 File). There was no appreciable overlap between the sets of genes showing differential expression and those affected by APA (Fig 5).

## Discussion

### The nature of the apicomplexan polyadenylation complex

The results of the bioinformatics study presented here raise interesting questions regarding the apicomplexan polyadenylation complex. Only two of the mammalian poly(A) complex subunits are clearly identifiable; these are the CPSF73 (or CPSF3) subunit and poly(A) polymerase. These two proteins arguably sit at the core of the polyadenylation reaction. CPSF73 is the endonuclease that processes the pre-mRNA [45, 46] and thus presents a substrate for PAP, the enzyme that adds the characteristic poly(A) tract to the 3’ end of the processed mRNA [2]. It is thus not surprising that these were easily identified in this analysis. In mammals and yeast, PAP is coordinated with the cleaved pre-mRNA by the CPSF subcomplex, and several protein-protein interactions between CPSF subunits and PAP have been reported; however, these do not include direct contacts between CPSF73 and PAP. Among other polyadenylation complex subunits that do engage in direct interactions with PAP are Fip1 [47–51], CPSF160 [52], CPSF100 [53, 54], and CFIm25 [49, 55]. Curiously, clear-cut orthologs of these proteins were not found in the three apicomplexan genomes studied here. PAP has been reported to interact with CPSF30 in some organisms [49]; the apicomplexan genomes studied here encode a possible CPSF30 ortholog, albeit one with somewhat limited sequence similarity with the human protein (panel F in S1 Fig). These considerations collectively raise interesting questions as to how the activities of the endonuclease (CPSF73) and PAP are coordinated in time and space. There may be functional counterparts to other PAP-interacting proteins that similarity searches cannot definitively identify. Alternatively, the CPSF-PAP complex in apicomplexans may be highly-reduced, such that only three proteins (CPSF73, PAP, and CPSF30) suffice for poly(A) signal recognition, pre-mRNA processing, and polyadenylation. These questions cannot be answered at the moment, but the disparate models represented have important implications for the functioning of the poly(A) complex, in the apicomplexans as well as other eukaryotes.

The matter of poly(A) signal recognition is of particular interest. The apicomplexan genomes studied in this report lack clear orthologs of one of the two subunits (WDR33) that associate with the motif AAUAAA in mammals, and they possess a possible ortholog of the other such subunit (CPSF30), albeit one with properties not seen in the mammalian counterpart. There does seem to be, in apicomplexans, a signal analogous to AAUAAA, and it reasonable to expect that the underlying mechanisms for recognition should be somewhat conserved. These considerations raise additional interesting questions. The apicomplexan genomes studied here possess genes that encode numerous WD repeat-containing proteins, any of which may be orthologs of WDR33. Alternatively, recognition of the A-rich PAS may not require a WDR33 ortholog, but instead could be performed by the CPSF30 ortholog. Plants are able to survive and thrive when CPSF30 is removed by mutation, providing precedent for the proposition that both WDR33 and CPSF30 are not required for mRNA 3’ end formation. This precedent makes somewhat more feasible the possibility that WDR33 orthologs may in fact be absent in apicomplexans. This possibility is further reinforced by the observation that clear orthologs of CPSF160, the scaffolding subunit upon which WDR33 and CPSF30 assemble in recognizing AAUAAA [42], are also not apparent in the apicomplexans; the possible absence of this subunit removes the means by which WDR33 and CPSF30 cooperate in the mammalian complex.

Perhaps the most provocative result of the bioinformatics study is the absence of identifiable orthologs of subunits of the Cleavage Stimulatory Factor (CstF) in the apicomplexans. This complex recognizes RNA sequences 3’ of the processing site [5], and plays important roles in processing and in alternative poly(A) site choice [7, 56, 57]. As is the case for WDR33, each of the three CstF subunits (CstF77, CstF64, and CstF50) possess motifs that are commonly found in proteins families. Also like WDR33, CstF50 possesses an array of WD repeats, and more than 30 WD repeat-containing proteins are found in *Plasmodium*, *Toxoplasma*, and *Sarcocystis* genome annotations. CstF64 possesses a distinctive RRM-type RNA-binding domain, and this domain is found in more than 40 proteins in different apicomplexan genome annotations. While the matches for any of these WDR and RRM proteins to CstF50 and CstF64 are rather tenuous, it remains possible that one or more may in fact serve analogous functions in apicomplexans. Surprisingly, however, even tenuous matches to CstF77 cannot be found in apicomplexan annotations, or when genomes are searched using TBLASTN. Given that CstF77 is the core scaffold upon which the other two subunits assemble to form a functional complex, this raises the intriguing possibility that mRNA 3’ end formation in apicomplexan species occurs without the participation of a CstF-like subcomplex.

### Stage-specific APA and gene regulation

As is the case in most eukaryotes, the majority of *S*. *neurona* genes possess more than one poly(A) site (S3 Fig); the median number of sites per gene is 3, and average is slightly greater than 5 (S4 File). Thus, the propensity for APA is great in this organism. It is thus not surprising that more than 200 genes are impacted by APA when comparing extracellular merozoites with intracellular schizonts. Interestingly, the analyses suggested that APA has a disproportionate impact on genes that encode ribosomal proteins and membrane transporters (S5 File, Table 2). For the most part, these impacts are such that the respective mRNAs have somewhat longer 3’-UTRs in merozoites (such as shown in Fig 4B), with no appreciable difference in overall transcript levels. This suggests the differing properties of the 3’-UTRs that dominate in the two developmental stages may play arole in posttranscriptional or translational control in the expression of these genes. Certainly, higher levels of ribosomal proteins and membrane transporters are consistent with the need for enhanced protein translation and nutrient acquisition, respectively, during intracellular development by schizonts. While these findings are preliminary and speculative, a role for APA in controlling protein levels during development of apicomplexan parasites seems plausible and warrants future investigation.

## Supporting information

S1 Fig Alignments of WDR33, PABN1, CFIm25 and CPSF30. Panels A-F: Amino acid sequence alignments of putative orthologs of CPSF73 (A), PAP (B), WDR33 (C), CFIm25 (D), PABN1 (E), and CPSF30 (or CPSF4; panel F). In all cases the human protein was aligned with three representative plant orthologs (from *Arabidopsis*, rice, and moss) as well as the closest matches (determined by BLASTP) found in the annotated genomes *of S*. *neurona*, *T*. *gondii*, and *P*. *falciparum*. Alignments were performed using the default setting in CLC Genomics Workbench. Sequences used in this alignment were:
Arabidopsis: AT1G61010 (CPSF73), AT1G17980 (PAP), At5g13480 (WDR33), AT4G25550 (CFIm25), AT5G10350 (PABN1), AT1g30460 (CPSF30-L)
Rice: LOC_Os03g63590 (CPSF73), Os06g21470 (PAP), Os01g72220 (WDR33), Os04g58640 (CFIm25), Os02g52140 (PABN1), Os06g46400 (CPSF30)
Moss (Physcomitrella patens): Pp1s23_196V6.1 (CPSF73), Pp1s3_426V6.1 (PAP), Pp1s197_75V6.1 (WDR33), Pp1s35_259V6.1 (CFIm25), Pp1s422_18V6.1 (PABN1), Pp1s9_445V6.1 (CPSF30)
Plasmodium falciparum: PF3D7_1438500 (CPSF73), PF3D7_0625600 (PAP), PF3D7_1241100 (WDR33), PF3D7_0109200 (CFIm25), PF3D7_0923900 (PABN1), PF3D7_1419900 (CPSF30)
Sarcocystis neurona: SN3_01500330 (CPSF73), SN3_00102700 (PAP), SN3_02200110 (WDR33), SN3_01200470 (CFIm25), SN3_00202270 (PABN1), SN3_00601130 (CPSF30)
Toxoplasma gondii: TGME49_285200-t26 (CPSF73), TGME49_226080-t26 (PAP), TGME49_268250-t26 (WDR33), TGME49_221190-t26 (CFIm25), TGME49_211020-t26 (PABN1), TGME49_201200-t26 (CPSF30)
Human: CPSF3, PAPa, WDR33, NUDT21, BCL212-PABPN1, CPSF4
For each alignment, the overall similarity across the seven sequences is depicted on the color bar, with red being the greatest sequence identity and blue the least. Amino acid residues that are identical in all seven sequences are shown in black, and other residues in gray. Notable domains (metallo-beta-lactamase conserved motifs, PAP catalytic residues, WD repeat, NUDIX, RRM, zinc finger, YTH) are highlighted in the respective panels. The alignments for CPSF73 and PAP were truncated, focusing on the conserved cores of the proteins. The alignment for WDR33 was split into two panels (C and C part 2) to facilitate viewing. Panel G: Confirmation of expression of the *S*. *neurona* gene that encodes a putative CPSF30-YTH protein. Portions of the coding regions (shaded in yellow) that encode the zinc finger and YTH domains are shown with the red and blue shaded boxes beneath the gene illustration for the RNA-Seq mapping.
(PDF)

S2 Fig Nucleotide composition surrounding genomic poly(A) tracts in three apicomplexan genomes. Nucleotide composition surrounding tracts of six or more A’s situated within 3’-untranslated regions were identified and the adjacent nucleotide compositions surrounding these plotted in similar fashion. Data points represent the relative fractional composition of each nucleotide at each position extending from 50 nts upstream to 50 nts downstream of the experimentally-determined or mock sites. The organism under study is indicated beneath each plot.
(PDF)

S3 Fig Number of poly(A) site clusters per gene in *S*. *neurona*. The numbers of PACs per gene were calculated using the data in S4 File and plotted as shown.
(PDF)

S1 File Summary of sequence libraries. Mapping and other statistics for each library generated in this study.
(XLSX)

S2 File Analysis of mRNA lengthening/shortening during development in *S*. *neurona*. Poly(A) site clusters in *S*. *neurona* were analyzed to determine shifts in average mRNA lengths.
(XLSX)

S3 File Gene expression summaries. Gene expression was determined by mapping poly(A) tags to individual *S*. *neurona* genes and the results used to assess differential gene expression.
(XLSX)

S4 File List of poly(A) sites in *S*. *neurona*. This sheet has a complete list of all S. neurona PACs that are defined by the poly(A) tag reads generated in this study.
(XLSX)

S5 File Analysis of differential poly(A) site usage in *S*. *neurona*. Poly(A) site clusters in *S*. *neurona* were tabulated and analyzed to identify PACs showing differential utilization at different growth stages.
(XLSX)

S1 Table Oligonucleotides used in this study. (DOCX)
