# Supplementary material for: Characterization of mRNA polyadenylation in the apicomplexa
Source: PLoS One. 2018 Aug 30;13(8):e0203317. doi: 10.1371/journal.pone.0203317 (PMC6117058; doi:10.1371/journal.pone.0203317)
Supplement: S1 Fig — Panels A-F: Amino acid sequence alignments of putative orthologs of CPSF73 (A), PAP (B), WDR33 (C), CFIm25 (D), PABN1 (E), and CPSF30 (or CPSF4; panel F). In all cases the human protein was aligned with three representative plant orthologs (from Arabidopsis, rice, and moss) as well as the closest matches (determined by BLASTP) found in the annotated genomes of S. neurona, T. gondii, and P. falciparum. Alignments were performed using the default setting in CLC Genomics Workbench. Sequences used in this alignment were: Arabidopsis: AT1G61010 (CPSF73), AT1G17980 (PAP), At5g13480 (WDR33), AT4G25550 (CFIm25), AT5G10350 (PABN1), AT1g30460 (CPSF30-L) Rice: LOC_Os03g63590 (CPSF73), Os06g21470 (PAP), Os01g72220 (WDR33), Os04g58640 (CFIm25), Os02g52140 (PABN1), Os06g46400 (CPSF30) Moss (Physcomitrella patens): Pp1s23_196V6.1 (CPSF73), Pp1s3_426V6.1 (PAP), Pp1s197_75V6.1 (WDR33), Pp1s35_259V6.1 (CFIm25), Pp1s422_18V6.1 (PABN1), Pp1s9_445V6.1 (CPSF30) Plasmodium falciparum: PF3D7_1438500 (CPSF73), PF3D7_0625600 (PAP), PF3D7_1241100 (WDR33), PF3D7_0109200 (CFIm25), PF3D7_0923900 (PABN1), PF3D7_1419900 (CPSF30) Sarcocystis neurona: SN3_01500330 (CPSF73), SN3_00102700 (PAP), SN3_02200110 (WDR33), SN3_01200470 (CFIm25), SN3_00202270 (PABN1), SN3_00601130 (CPSF30) Toxoplasma gondii: TGME49_285200-t26 (CPSF73), TGME49_226080-t26 (PAP), TGME49_268250-t26 (WDR33), TGME49_221190-t26 (CFIm25), TGME49_211020-t26 (PABN1), TGME49_201200-t26 (CPSF30) Human: CPSF3, PAPa, WDR33, NUDT21, BCL212-PABPN1, CPSF4 For each alignment, the overall similarity across the seven sequences is depicted on the color bar, with red being the greatest sequence identity and blue the least. Amino acid residues that are identical in all seven sequences are shown in black, and other residues in gray. Notable domains (metallo-beta-lactamase conserved motifs, PAP catalytic residues, WD repeat, NUDIX, RRM, zinc finger, YTH) are highlighted in the respective panels. The alignments for CPSF73 and PAP were truncated, focu [file pone.0203317.s001.pdf]

A

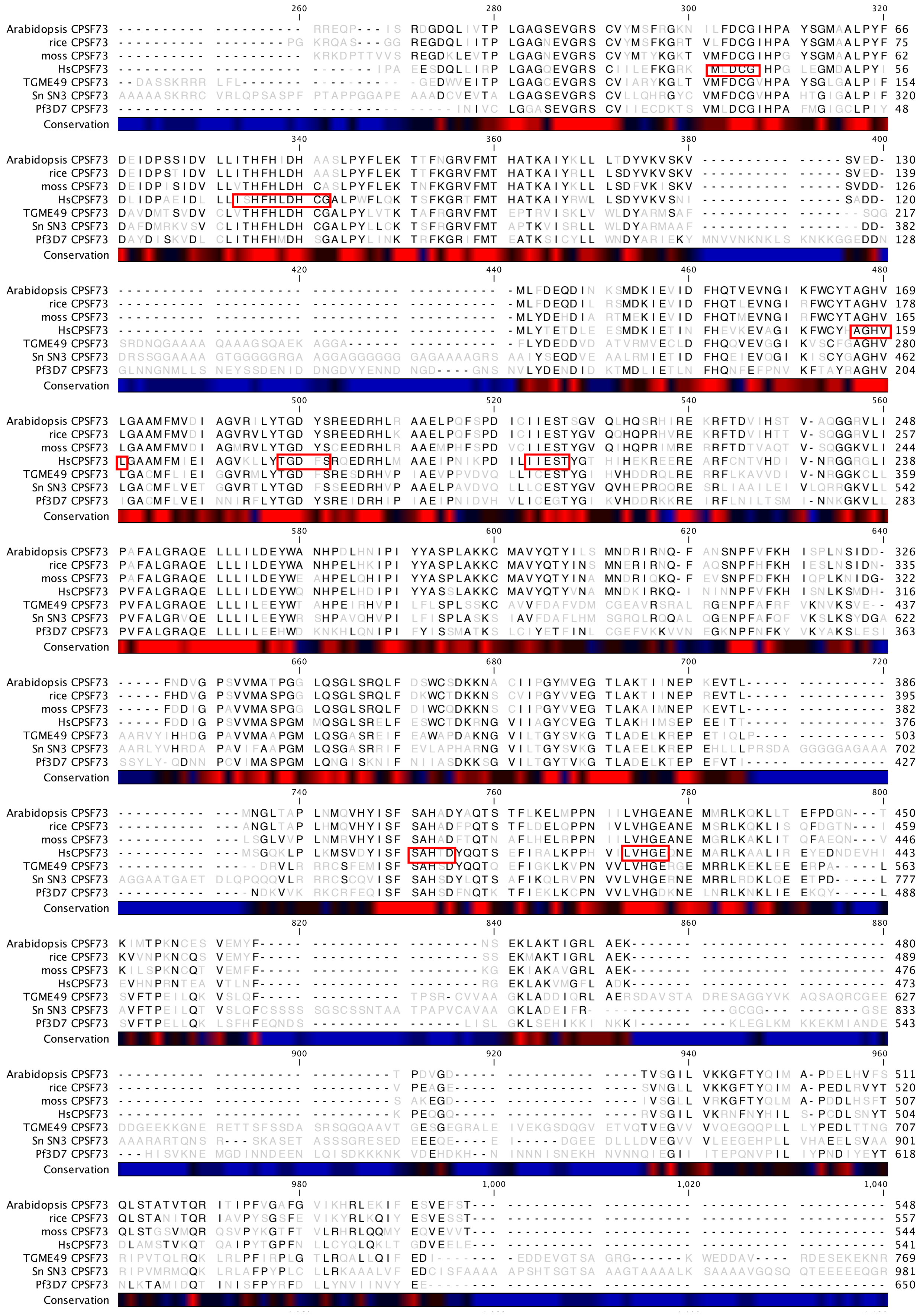

260 280 300 320
Arabidopsis CPSF73 ---------- ---------- RREQP---IS RDGDQLIVTP LGAGSEVGRS CVYMSFRGKN ILFDCGIHPA YSGMAALPYF 66
rice CPSF73 ---------- --------PG KRQAS---GG REGDQLIITP LGAGNEVGRS CVYMSFKGRT VLFDCGIHPA YSGMAALPYF 75
moss CPSF73 ---------- ---------- KRKDPTTVVS REGDKLEVTP LGAGNEVGRS CVYMTYKGKT VMFDCGIHPG YSGMAALPYF 62
HsCPSF73 ---------- ---------- -------IPA EESDQLLIRP LGAGQEVGRS CIILEFKGRK IMLDCGIHPG LEGMDALPYI 56
TGME49 CPSF73 --DASSKRRR LFL------- ---------- -GEDWVEITP LGAGCEVGRS CVIARYKGLT VMFDCGVHPA YSGLGALPIF 154
Sn SN3 CPSF73 AAAAASKRRC VRLQPSASPF PTAPPGGAPE AAADCVEVTA LGAGSEVGRS CVLLQHRGYC VMFDCGVHPA HTGIGALPIF 320
Pf3D7 CPSF73 ---------- ---------- ---------- -----INIVC LGGASEVGRS CVIIECDKTS VMLDCGIHPA FMGIGCLPIY 48
Conservation
340 360 380 400
Arabidopsis CPSF73 DEIDPSSIDV LLITHFHIDH AASLPYFLEK TTFNGRVFMT HATKAIYKLL LTDYVKVSKV ---------- -----SVED- 130
rice CPSF73 DEIDPSTIDV LLITHFHLDH AASLPYFLEK TTFKGRVFMT HATKAIYKLL LSDYVKVSKV ---------- -----SVED- 139
moss CPSF73 DEIDPISIDV LLVTHFHLDH CASLPYFLEK TNFKGRVFMT HATKAIYKLL LSDFVKISKV ---------- -----SVDD- 126
HsCPSF73 DLIDPAEIDL LLISHFHLDH CGALPWFLQK TSFKGRTFMT HATKAIYRWL LSDYVKVSNI ---------- -----SADD- 120
TGME49 CPSF73 DAVDMTSVDV CLVTHFHLDH CGALPYLVTK TAFRGRVFMT EPTRVISKLV WLDYARMSAF ---------- -------SQG 217
Sn SN3 CPSF73 DAFDMRKVSV CLITHFHLDH CGALPYLLCK TSFRGRVFMT APTKVISRLL WLDYARMAAF ---------- -------DD- 382
Pf3D7 CPSF73 DAYDISKVDL CLITHFHMDH SGALPYLINK TRFKGRIFMT EATKSICYLL WNDYARIEKY MNVVNKNKLS KNKKGGEDDN 128
Conservation
420 440 460 480
Arabidopsis CPSF73 ---------- ---------- ---------- ---------- -MLFDEQDIN KSMDKIEVID FHQTVEVNGI KFWCYTAGHV 169
rice CPSF73 ---------- ---------- ---------- ---------- -MLFDEQDIL RSMDKIEVID FHQTLEVNGI RFWCYTAGHV 178
moss CPSF73 ---------- ---------- ---------- ---------- -MLYDEHDIA RTMEKIEVID FHQTMEVNGI RFWCYTAGHV 165
HsCPSF73 ---------- ---------- ---------- ---------- -MLYTETDLE ESMDKIETIN FHEVKEVAGI KFWCYHAGHV 159
TGME49 CPSF73 SRDNQGAAAA QAAAGSQAEK AGGA------ ---------- -FLYDEDDVD ATVRMVECLD FHQQVEVGGI KVSCFGAGHV 280
Sn SN3 CPSF73 DRSSGGAAAA GTGGGGGRGA AGGAGGGGGG GAGAAAAGRS AAIYSEQDVE AALRMIETID FHQEIEVQGI KISCYGAGHV 462
Pf3D7 CPSF73 GLNNGNMLLS NEYSSDENID DNGDVYENND NGD----GNS NVLYDENDID KTMDLIETLN FHQNFEFPNV KFTAYRAGHV 204
Conservation
500 520 540 560
Arabidopsis CPSF73 LGAAMFMVDI AGVRILYTGD YSREEDRHLR AAELPQFSPD ICIIESTSGV QLHQSRHIRE KRFTDVIHST V-AQGGRVLI 248
rice CPSF73 LGAAMFMVDI AGVRVLYTGD YSREEDRHLK AAELPQFSPD ICIIESTYGV QQHQPRHVRE KRFTDVIHTT V-SQGGRVLI 257
moss CPSF73 LGAAMFMVDI AGMRVLYTGD YSCEEDRHLR AAEMPHFSPD VCIIESTYGV QIHQPRIMRE RRFTDTVAQT V-SQGGKVLI 244
HsCPSF73 LGAAMFMIEI AGVKLLYTGD FSRQEDRHLM AAEIPNIKPD ILIIESTYGT HIHEKREERE ARFCNTVHDI V-NRGGRGLI 238
TGME49 CPSF73 LGACMFLIEI GGVRMLYTGD FSRESDRHVP IAEVPPVDVQ LLICESTYGI HVHDDRQLRE RRFLKAVVDI V-NRGGKCLL 359
Sn SN3 CPSF73 LGACMFLVET GGVRTLYTGD FSSEEDRHVP AAELPAVDVQ LLLCESTYGV QVHEPRQQRE SRLIAAILEI VLQRRGKVLL 542
Pf3D7 CPSF73 IGACMFLVEI NNIRFLYTGD YSREIDRHIP IAEIPNIDVH VLICEGTYGI KVHDDRKKRE IRFLNILTSM I-NNKGKVLL 283
Conservation
580 600 620 640
Arabidopsis CPSF73 PAFALGRAQE LLLILDEYWA NHPDLHNIPI YYASPLAKKC MAVYQTYILS MNDRIRNQ-F ANSNPFVFKH ISPLNSIDD- 326
rice CPSF73 PAFALGRAQE LLLILDEYWA NHPELHKIPI YYASPLAKKC MAVYQTYINS MNERIRNQ-F AQSNPFHFKH IESLNSIDN- 335
moss CPSF73 PAFALGRAQE LLLILDEYWE AHPELQHIPI YYASPLAKKC MAVYQTYINA MNDRIQKQ-F EVSNPFDFKH IQPLKNIDG- 322
HsCPSF73 PVFALGRAQE LLLILDEYWQ NHPELHDIPI YYASSLAKKC MAVYQTYVNA MNDKIRKQ-I NINNPFVFKH ISNLKSMDH- 316
TGME49 CPSF73 PVFALGRAQE LLLILEEYWT AHPEIRHVPI LFLSPLSSKC AVVFDAFVDM CGEAVRSRAL RGENPFAFRF VKNVKSVE-- 437
Sn SN3 CPSF73 PVFALGRVQE LLLILEEYWR SHPAVQHVPI LFISPLASKS IAVFDAFLHM SGRQLRQQAL QGENPFAFQF VKSLKSYDGA 622
Pf3D7 CPSF73 PVFALGRAQE LLLILEEHWD KNKHLQNIPI FYISSMATKS LCIYETFINL CGEFVKKVVN EGKNPFNFKY VKYAKSLESI 363
Conservation
660 680 700 720
Arabidopsis CPSF73 -----FNDVG PSVVMATPGG LQSGLSRQLF DSWCSDKKNA CIIPGYMVEG TLAKTIINEP KEVTL----- ---------- 386
rice CPSF73 -----FHDVG PSVVMASPGG LQSGLSRQLF DKWCTDKKNS CVIPGYVVEG TLAKTIINEP REVTL----- ---------- 395
moss CPSF73 -----FDDIG PAVVMASPGG LQSGLSRQLF DIWCGDKKNS CIIPGYVVEG TLAKAIMNEP KEVTL----- ---------- 382
HsCPSF73 -----FDDIG PSVVMASPGM MQSGLSRELF ESWCTDKRNG VIIAGYCVEG TLAKHIMSEP EEITT----- ---------- 376
TGME49 CPSF73 AARVYIHHDG PAVVMAAPGM LQSGASREIF EAWAPDAKNG VILTGYSVKG TLADELKREP ETIQLP---- ---------- 503
Sn SN3 CPSF73 AARLYVHRDA PAVIFAAPGM LQSGASRRIF EVLAPHARNG VILTGYSVKG TLAEELKREP EHLLLPRSDA GGGGGAGAAA 702
Pf3D7 CPSF73 SSYLY-QDNN PCVIMASPGM LQNGISKNIF NIIASDKKSG VILTGYTVKG TLADELKTEP EFVTI----- ---------- 427
Conservation
740 760 780 800
Arabidopsis CPSF73 ---------- ---MNGLTAP LNMQVHYISF SAHADYAQTS TFLKELMPPN IILVHGEANE MMRLKQKLLT EFPDGN---T 450
rice CPSF73 ---------- ---ANGLTAP LHMQVHYISF SAHADFPQTS TFLDELQPPN IVLVHGEANE MSRLKQKLIS QFDGTN---I 459
moss CPSF73 ---------- ---LSGLVVP LNMRVHYISF SAHADFTQTN AFLHELRPPN IILVHGEANE MGRLKAKLIT QFAEQN---V 446
HsCPSF73 ---------- ---MSGQKLP LKMSVDYISF SAHTDYQQTS EFIRALKPPH VILVHGEQNE MARLKAALIR EYEDNDEVHI 443
TGME49 CPSF73 ---------- -----DRVLR RRCSFEMISF SAHSDYQQTQ EFIGKLKVPN VVLVHGERGE MRRLKEKLEE ERPA-----L 563
Sn SN3 CPSF73 AGGAATGAET DLQPQQQVLR RRCSCQVISF SAHSDYLQTS AFIKQLRVPN VVLVHGERNE MRRLRDKLQE ETPD-----L 777
Pf3D7 CPSF73 ---------- ----NDKVVK RKCRFEQISF SAHSDFNQTK TFIEKLKCPN VVLVHGDKNE LNRLKNKLIE EKQY-----L 488
Conservation
820 840 860 880
Arabidopsis CPSF73 KIMTPKNCES VEMYF----- ---------- ---------NS EKLAKTIGRL AEK------- ---------- ---------- 480
rice CPSF73 KVVNPKNCQS VEMYF----- ---------- ---------SS EKMAKTIGRL AEK------- ---------- ---------- 489
moss CPSF73 KILSPKNCQT VEMFF----- ---------- --------KG EKIAKAVGRL AEK------- ---------- ---------- 476
HsCPSF73 EVHNPRNTEA VTLNF----- ---------- --------RG EKLAKVMGFL ADK------- ---------- ---------- 473
TGME49 CPSF73 SVFTPEILQK VSLQF----- ---------- TPSR-CVKAA GKLADDIQRL AERSDAVSTA DRESAGGYVK AQSAQRCGEE 627
Sn SN3 CPSF73 AVFTPEILQT VSLQFCSSSS SGSCSSNTAA TPAPVCAVAA GKLADEIFR- ----GCGG-- -------GSE 833
Pf3D7 CPSF73 SVFTPELLQK LSFHFEQNDS ---------- ------LISL GKLSEHIKKI NKKI------ ---KLEGLKM KKEKMIANDE 543
Conservation
900 920 940 960
Arabidopsis CPSF73 ---------- ---------- ---------T PDVGD----- ---------- ----TVSGIL VKKGFTYQIM A-PDELHVFS 511
rice CPSF73 ---------- ---------- ---------V PEAGE----- ---------- ----SVNGLL VKKGFTYQIM A-PEDLRVYT 520
moss CPSF73 ---------- ---------- ---------S AKEGD----- ---------- ----IVSGLL VRKGFTYQLM A-PDDLHSFT 507
HsCPSF73 ---------- ---------- ---------K PEGQ------ ---------- ----RVSGIL VKRNFNYHIL S-PCDLSNYT 504
TGME49 CPSF73 DDGEEKKGNE RETTSFSSDA SRSQGQAAVT GESGEGRALE IVEKGSDQGV ETVQTVEGVV VVQEGQQPLL LYPEDLTTNG 707
Sn SN3 CPSF73 AAARARTQNS R---SKASET ASSSGRESED EEEQE----E I-----DGEE DLLLDVEGVV VLEEGEHPLL VHAEELSVAA 901
Pf3D7 CPSF73 ---HISVKNE MGDINNDEEN LQISDKKKNK VDEHDKH--N INNNISNEKH NVNNQIEGII ITEPQNVPIL IYPNDIYEYT 618
Conservation
980 1,000 1,020 1,040
Arabidopsis CPSF73 QLSTATVTQR ITIPFVGAFG VIKHRLEKIF ESVEFST--- ---------- ---------- ---------- ---------- 548
rice CPSF73 QLSTANITQR IAVPYSGSFE VIKYRLKQIY ESVESST--- ---------- ---------- ---------- ---------- 557
moss CPSF73 QLSTGSVMQR QSVPYKGTFT VLRHRLQQMY EQVEVVT--- ---------- ---------- ---------- ---------- 544
HsCPSF73 DLAMSTVKQT QAIPYTGPFN LLCYQLQKLT GDVEELE--- ---------- ---------- ---------- ---------- 541
TGME49 CPSF73 RIPVTQLRQK LRLPFIRPLG TLRQALLQIF EDI------- EDDEVGTSA GRG------K WEDDAV---- RDESEKEKNR 769
Sn SN3 CPSF73 RIPVMRMQQK LRLAFPYPLC LLRKAAALVF EDCISFAAAA APSHTSGTSA AAGTAAAALK SAAAAVGQSQ QTEEEEEQGR 981
Pf3D7 CPSF73 NLKTAMIDQT INISFPYRFD LLYNVIINVY EE-------- ---------- ---------- ---------- ---------- 650
Conservation

B

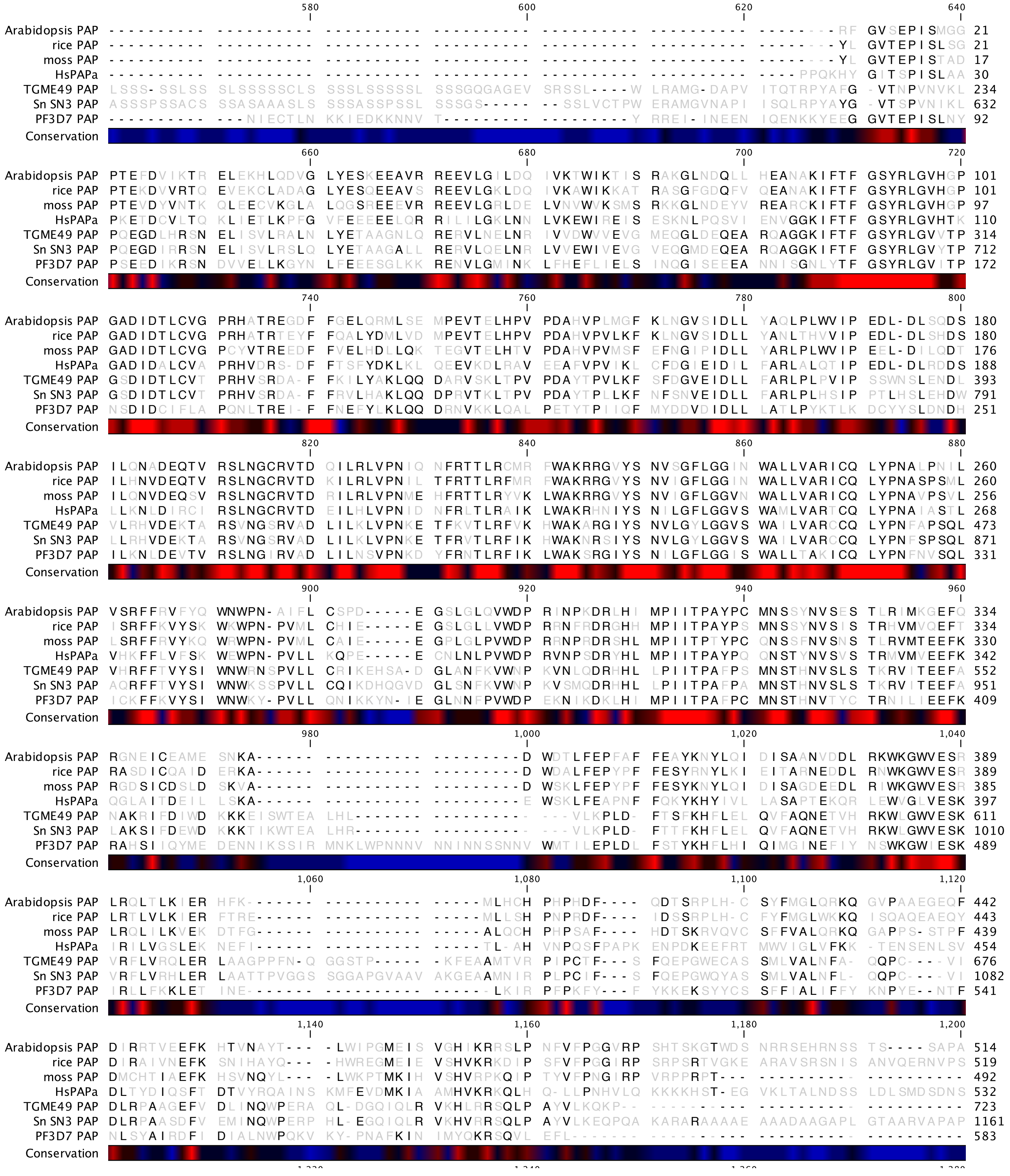
580 600 620 640
Arabidopsis PAP ---------- ---------- ---------- ---------- ---------- ---------- --------RF GVSEPISMGG 21
rice PAP ---------- ---------- ---------- ---------- ---------- ---------- ---------YL GVTEPISLSG 21
moss PAP ---------- ---------- ---------- ---------- ---------- ---------- ---------YL GVTEPISTAD 17
HsPAPa ---------- ---------- ---------- ---------- ---------- ---------- ----PPQKHY GITSPISLAA 30
TGME49 PAP LSSS-SSLSS SLSSSSSCLS SSSLSSSSSL SSSGQGAGEV SRSSL----W LRAMG-DAPV ITQTRPYAFG -VTNPVNVKL 234
Sn SN3 PAP ASSSPSSACS SSASAAASLS SSSASSPSSL SSSGS----- --SSLVCTPW ERAMGVNAPI ISQLRPYAYG -VTSPVNIKL 632
PF3D7 PAP ---------- ---NIECTLN KKIEDKKNNV T--------- ---------Y RREI-INEEN IQENKKYEEG GVTEPISLNY 92
Conservation
660 680 700 720
Arabidopsis PAP PTEFDVIKTR ELEKHLQDVG LYESKEEAVR REEVLGILDQ IVKTWIKTIS RAKGLNDQLL HEANAKIFTF GSYRLGVHGP 101
rice PAP PTEKDVVRTQ EVEKCLADAG LYESQEEAVS REEVLGKLDQ IVKAWIKKAT RASGFGDQFV QEANAKIFTF GSYRLGVHGP 101
moss PAP PTEVDYVNTK QLEECVKGLA LQGSREEEVR REEVLGRLDE LVNVWVKSMS RKKGLNDEYV REARCKIFTF GSYRLGVHGP 97
HsPAPa PKETDCVLTQ KLIETLKPFG VFEEEEELQR RILILGKLNN LVKEWIREIS ESKNLPQSVI ENVGGKIFTF GSYRLGVHTK 110
TGME49 PAP PQEGDLHRSN ELISVLRALN LYETAAGNLQ RERVLNELNR IVVDWVVEVG MEQGLDEQEA RQAGGKIFTF GSYRLGVVTP 314
Sn SN3 PAP PQEGDIRRSN ELISVLRSLQ LYETAAGALL RERVLQELNR LVVEWIVEVG VEQGMDEQEA RQAGGKIFTF GSYRLGVYTP 712
PF3D7 PAP PSEEDIKRSN DVVELLKGYN LFEEESGLKK RENVLGMINK LFHEFLIELS INQGISEEEA NNISGNLYTF GSYRLGVITP 172
Conservation
740 760 780 800
Arabidopsis PAP GADIDTLCVG PRHATREGDF FGELQRMLSE MPEVTELHPV PDAHVPLMGF KLNGVSIDLL YAQLPLWVIP EDL-DLSQDS 180
rice PAP GADIDTLCVG PRHATRTEYF FQALYDMLVD MPEVTELHPV PDAHVPVLKF KLNGVSIDLL YANLTHVVIP EDL-DLSHDS 180
moss PAP GADIDTLCVG PCYVTREEDF FVELHDLLQK TEGVTELHTV PDAHVPVMSF EFNGIPIDLL YARLPLWVIP EEL-DILQDT 176
HsPAPa GADIDALCVA PRHVDRS-DF FTSFYDKLKL QEEVKDLRAV EEAFVPVIKL CFDGIEIDIL FARLALQTIP EDL-DLRDDS 188
TGME49 PAP GSDIDTLCVT PRHVSRDA-F FKILYAKLQQ DARVSKLTPV PDAYTPVLKF SFDGVEIDLL FARLPLPVIP SSWNSLENDL 393
Sn SN3 PAP GSDIDTLCVT PRHVSRDA-F FRVLHAKLQQ DPRVTKLTPV PDAYTPLLKF NFSNVEIDLL FARLPLHSIP PTLHSLEHDW 791
PF3D7 PAP NSDIDCIFLA PQNLTREI-F FNEFYLKLQQ DRNVKKLQAL PETYTPIIQF MYDDVDIDLL LATLPYKTLK DCYYSLDNDH 251
Conservation
820 840 860 880
Arabidopsis PAP ILQNADEQTV RSLNGCRVTD QILRLVPNIQ NFRTTLRCMR FWAKRRGVYS NVSGFLGGIN WALLVARICQ LYPNALPNIL 260
rice PAP ILHNVDEQTV RSLNGCRVTD KILRLVPNIL TFRTTLRFMR FWAKRRGVYS NVIGFLGGIN WALLVARICQ LYPNASPSML 260
moss PAP ILQNVDEQSV RSLNGCRVTD RILRLVPNME HFRTTLRYVK LWAKRRGVYS NVIGFLGGVN WALLVARICQ LYPNAVPSVL 256
HsPAPa LLKNLDIRCI RSLNGCRVTD EILHLVPNID NFRLTLRAIK LWAKRHNIYS NILGFLGGVS WAMLVARTCQ LYPNAIASTL 268
TGME49 PAP VLRHVDEKTA RSVNGSRVAD LILKLVPNKE TFKVTLRFVK HWAKARGIYS NVLGYLGGVS WAILVARCCQ LYPNFAPSQL 473
Sn SN3 PAP LLRHVDEKTA RSVNGSRVAD LILKLVPNKE TFRVTLRFIK HWAKNRSIYS NVLGYLGGVS WAILVARCCQ LYPNFSPSQL 871
PF3D7 PAP ILKNLDEVTV RSLNGIRVAD LILNSVPNKD YFRNTLRFIK LWAKSRGIYS NILGFLGGIS WALLTAKICQ LYPNFNVSQL 331
Conservation
900 920 940 960
Arabidopsis PAP VSRFFRVFYQ WNWPN-AIFL CSPD-----E GSLGLQVWDP RINPKDRLHI MPIITPAYPC MNSSYNVSES TLRIMKGEFQ 334
rice PAP ISRFFKVYSK WKWPN-PVML CHIE-----E GSLGLLVWDP RRNFRDRGHH MPIITPAYPS MNSSYNVSIS TRHVMVQEFT 334
moss PAP LSRFFRVYKQ WRWPN-PVML CAIE-----E GPLGLPVWDP RRNPRDRSHL MPIITPTYPC QNSSFNVSNS TLRVMTEEFK 330
HsPAPa VHKFFLVFSK WEWPN-PVLL KQPE-----E CNLNLPVWDP RVNPSDRYHL MPIITPAYPQ QNSTYNVSVS TRMVMVEEFK 342
TGME49 PAP VHRFFTVYSI WNWRNSPVLL CRIKEHSA-D GLANFKVWNP KVNLQDRHHL LPIITPAFPS MNSTHNVSLS TKRVITEEFA 552
Sn SN3 PAP AQRFFTVYSI WNWKSSPVLL CQIKDHQGVD GLSNFKVWNP KVSMQDRHHL LPIITPAFPA MNSTHNVSLS TKRVITEEFA 951
PF3D7 PAP ICKFFKVYSI WNWKY-PVLL QNIKKYN-IE GLNNFPVWDP EKNIKDKLHI MPIITPAFPC MNSTHNVTYC TRNILIEEFK 409
Conservation
980 1,000 1,020 1,040
Arabidopsis PAP RGNEICEAME SNKA------ ---------- ---------D WDTLFEPFAF FEAYKNYLQI DISAANVDDL RKWKGWVESR 389
rice PAP RASDICQAID ERKA------ ---------- ---------D WDALFEPYPF FESYRNYLKI EITARNEDDL RNWKGWVESR 389
moss PAP RGDSICDSLD SKVA------ ---------- ---------D WSKLFEPYPF FESYKNYLQI DISAGDEEDL RIWKGWVESR 385
HsPAPa QGLAITDEIL LSKA------ ---------- ---------E WSKLFEAPNF FQKYKHYIVL LASAPTEKQR LEWVGLVESK 397
TGME49 PAP NAKRIFDIWD KKKEISWTEA LHL------- ---------- ---VLKPLD- FTSFKHFLEL QVFAQNETVH RKWLGWVESK 611
Sn SN3 PAP LAKSIFDEWD KKKTIKWTEA LHR------- ---------- ---VLKPLD- FTTFKHFLEL QVFAQNETVH RKWLGWVESK 1010
PF3D7 PAP RAHSIIQYME DENNIKSSIR MNKLWPNNNV NNINNSSNNV WMTILEPLDL FSTYKHFLHI QIMGINEFIY NSWKGWIESK 489
Conservation
1,060 1,080 1,100 1,120
Arabidopsis PAP LRQLTLKIER HFK------- ---------- -----MLHCH PHPHDF---- QDTSRPLH-C SYFMGLQRKQ GVPAAEGEQF 442
rice PAP LRTLVLKIER FTRE------ ---------- -----MLLSH PNPRDF---- IDSSRPLH-C FYFMGLWKKQ ISQAQEAEQY 443
moss PAP LRQLILKVEK DTFG------ ---------- -----ALQCH PHPSAF---- HDTSKRVQVC SFFVALQRKQ GAPPS-STPF 439
HsPAPa IRILVGSLEK NEFI------ ---------- -----TL-AH VNPQSFPAPK ENPDKEEFRT MWVIGLVFKK -TENSENLSV 454
TGME49 PAP VRFLVRQLER LAAGPPFN-Q GGSTP----- -KFEAAMTVR PIPCTF---S FQEPGWECAS SMLVALNFA- QQPC----VI 676
Sn SN3 PAP VRFLVRHLER LAATTPVGGS SGGAPGVAAV AKGEAAMNIR PLPCIF---S FQEPGWQYAS SMLVALNFL- QQPC----VI 1082
PF3D7 PAP IRLLFKKLET INE------- ---------- ------LKIR PFPKFY---F YKKEKSYYCS SFFIALIFFY KNPYE--NTF 541
Conservation
1,140 1,160 1,180 1,200
Arabidopsis PAP DIRRTVEEFK HTVNAYT--- -LWIPGMEIS VGHIKRRSLP NFVFPGGVRP SHTSKGTWDS NRRSEHRNSS TS----SAPA 514
rice PAP DIRAIVNEFK SNIHAYQ--- -HWREGMEIE VSHVKRKDIP SFVFPGGIRP SRPSRTVGKE ARAVSRSNIS ANVQERNVPS 519
moss PAP DMCHTIAEFK HSVNQYL--- -LWKPTMKIH VSHVRPKQIP TYVFPNGIRP VRPPRPT--- ---------- ---------- 492
HsPAPa DLTYDIQSFT DTVYRQAINS KMFEVDMKIA AMHVKRKQLH Q-LLPNHVLQ KKKKHST-EG VKLTALNDSS LDLSMDSDNS 532
TGME49 PAP DLRPAAGEFV DLINQWPERA QL-DGQIQLR VKHLRRSQLP AYVLKQKP-- ---------- ---------- ---------- 723
Sn SN3 PAP DLRPAASDFV EMINQWPERP HL-EGQIQLR VKHVRRSQLP AYVLKEQPQA KARARAAAAE AAADAAGAPL GTAARVAPAP 1161
PF3D7 PAP NLSYAIRDFI DIALNWPQKV KY-PNAFKIN IMYQKRSQVL EFL------- ---------- ---------- ---------- 583
Conservation

C

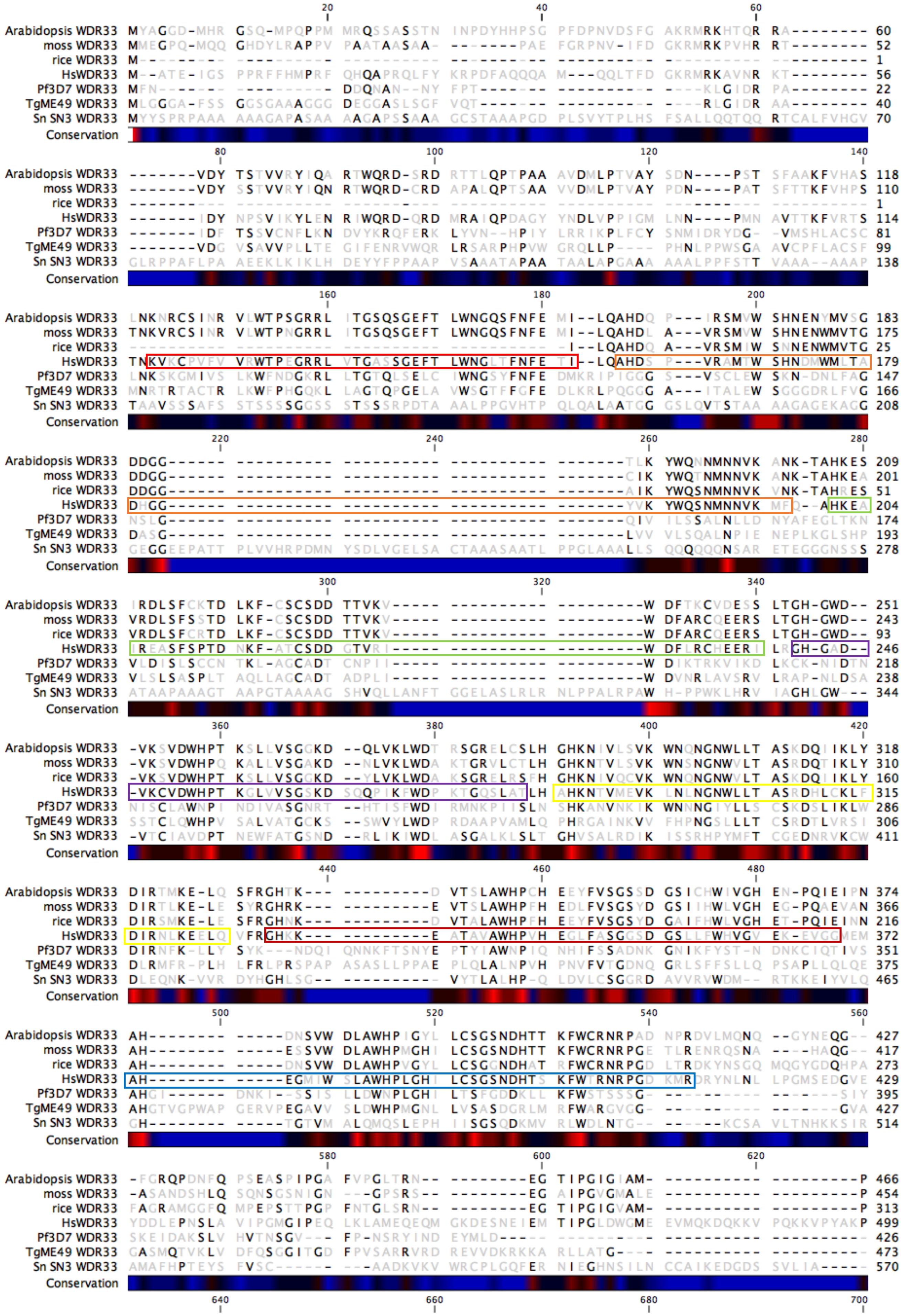
Arabidopsis WDR33
moss WDR33
rice WDR33
HsWDR33
Pf3D7 WDR33
TgME49 WDR33
Sn SN3 WDR33
Conservation

C (part 2)

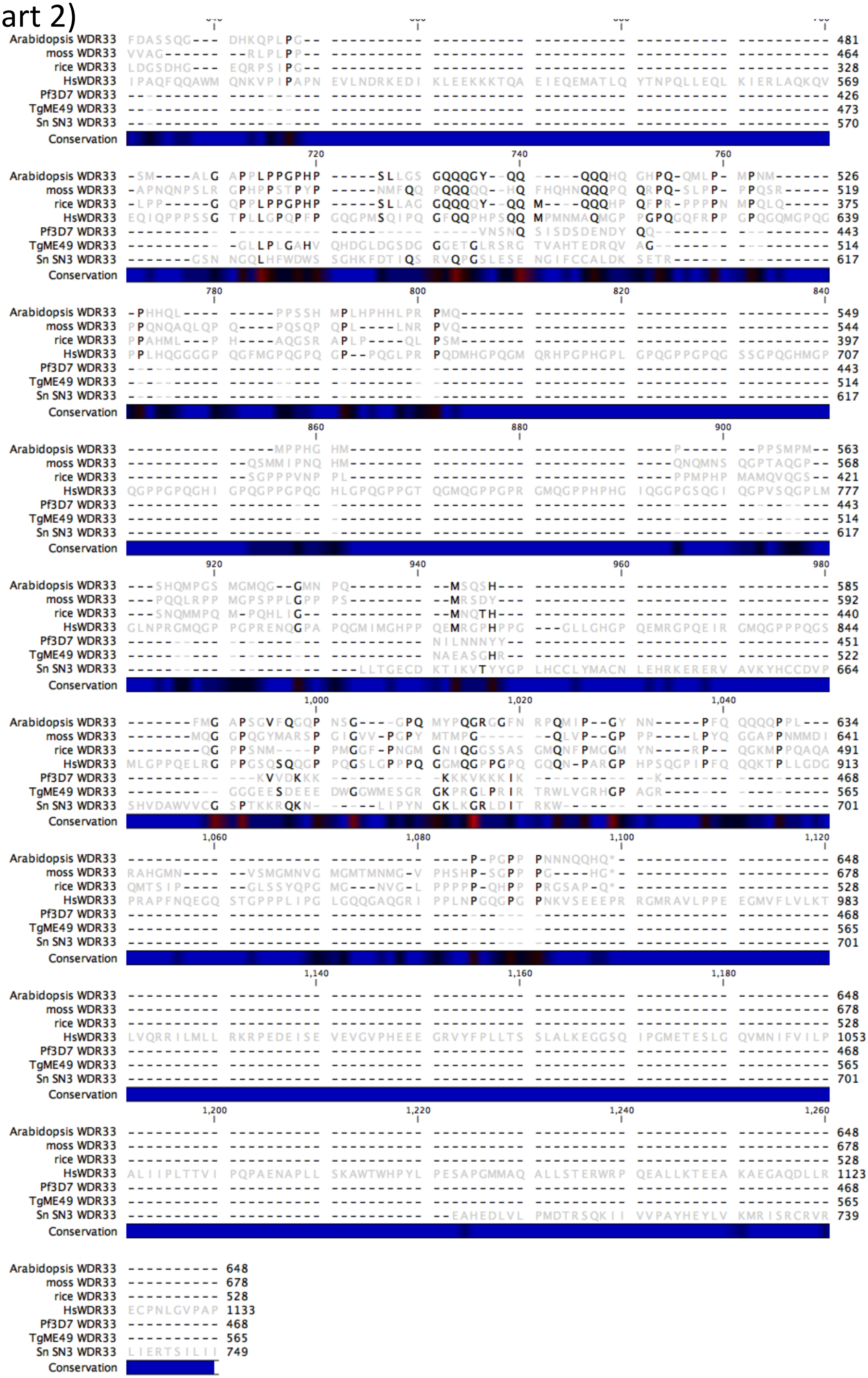
Arabidopsis WDR33 FDASSQG--- DHKQPLPG-- ---------- ---------- ---------- ---------- ---------- 481
moss WDR33 VVAG------ --RLPLPP-- ---------- ---------- ---------- ---------- ---------- 464
rice WDR33 LDGSDHG--- EQRPSIPG-- ---------- ---------- ---------- ---------- ---------- 328
HsWDR33 IPAQFQQAWM QNKVPIPAPN EVLNDRKEDI KLEEKKKTQA EIEQEMATLQ YTNPQLLEQL KIERLAQKQV 569
Pf3D7 WDR33 ---------- ---------- ---------- ---------- ---------- ---------- ---------- 426
TgME49 WDR33 ---------- ---------- ---------- ---------- ---------- ---------- ---------- 473
Sn SN3 WDR33 ---------- ---------- ---------- ---------- ---------- ---------- ---------- 570
Conservation
720 740 760
Arabidopsis WDR33 -SM----ALG APPLPPGPHP -----SLLGS GQQQGY--QQ -----QQQHQ GHPQ-QMLP- MPNM------ 526
moss WDR33 -APNQNPSLR GPHPPSTPYP -----NMFQQ PQQQQQ--HQ FHQHNQQQPQ QRPQ-SLPP- PPQSR----- 519
rice WDR33 -LPP-----G QPPLPPGPHP -----SLLAG GQQQQY--QQ M----QQQHP QFPR-PPPPN MPQLQ----- 375
HsWDR33 EQIQPPPSSG TPLLGPQPFP GQGPMSQIPQ GFQQPHPSQQ MPMNMAQMGP PGPQGQFRPP GPQGQMGPQG 639
Pf3D7 WDR33 ---------- ---------- ---------- -----VNSNQ SISDSDENDY QQ-------- ---------- 443
TgME49 WDR33 ---------- -GLLPLGAHV QHDGLDGSDG GGETGLRSRG TVAHTEDRQV AG-------- ---------- 514
Sn SN3 WDR33 -------GSN NGQLHFWDWS SGHKFDTIQS RVQPGSLESE NGIFCCALDK SETR------ ---------- 617
Conservation
780 800 820 840
Arabidopsis WDR33 -PHHQL---- -----PPSSH MPLHPHHLPR PMQ------- ---------- ---------- ---------- 549
moss WDR33 PPQNQAQLQP Q----PQSQP QPL----LNR PVQ------- ---------- ---------- ---------- 544
rice WDR33 PPAHML---P H----AQGSR APLP----QL PSM------- ---------- ---------- ---------- 397
HsWDR33 PPLHQGGGGP QGFMGPQGPQ GP--PQGLPR PQDMHGPQGM QRHPGPHGPL GPQGPPGPQG SSGPQGHMGP 707
Pf3D7 WDR33 ---------- ---------- ---------- ---------- ---------- ---------- ---------- 443
TgME49 WDR33 ---------- ---------- ---------- ---------- ---------- ---------- ---------- 514
Sn SN3 WDR33 ---------- ---------- ---------- ---------- ---------- ---------- ---------- 617
Conservation
860 880 900
Arabidopsis WDR33 ---------- -----MPPHG HM-------- ---------- ---------- ----P----- --PPSMPM-- 563
moss WDR33 ---------- --QSMMIPNQ HM-------- ---------- ---------- ----QNQMNS QGPTAQGP-- 568
rice WDR33 ---------- --SGPPPVNP PL-------- ---------- ---------- ----PPMPHP MAMQVQGS-- 421
HsWDR33 QGPPGPQGHI GPQGPPGPQG HLGPQGPPGT QGMQGPPGPR GMQGPPHPHG IQGGPGSQGI QGPVSQGPLM 777
Pf3D7 WDR33 ---------- ---------- ---------- ---------- ---------- ---------- ---------- 443
TgME49 WDR33 ---------- ---------- ---------- ---------- ---------- ---------- ---------- 514
Sn SN3 WDR33 ---------- ---------- ---------- ---------- ---------- ---------- ---------- 617
Conservation
920 940 960 980
Arabidopsis WDR33 ---SHQMPGS MGMQG--GMN PQ-------- --MSQSH--- ---------- ---------- ---------- 585
moss WDR33 ---PQQLRPP MGPSPPLGPP PS-------- --MRSDY--- ---------- ---------- ---------- 592
rice WDR33 ---SNQMMPQ M-PQHLIG-- ---------- --MNQTH--- ---------- ---------- ---------- 440
HsWDR33 GLNPRGMQGP PGPRENQGPA PQGMIMGHPP QEMRGPHPPG ---GLLGHGP QEMRGPQEIR GMQGPPPQGS 844
Pf3D7 WDR33 ---------- ---------- ---------- NILNNNYY-- ---------- ---------- ---------- 451
TgME49 WDR33 ---------- ---------- ---------- NAEASGHR-- ---------- ---------- ---------- 522
Sn SN3 WDR33 ---------- ---------- ---LLTGECD KTIKVTYYGP LHCCLYMACN LEHRKERERV AVKYHCCDVP 664
Conservation
1,000 1,020 1,040
Arabidopsis WDR33 -------FMG APSGVFQGQP NSG----GPQ MYPQGRGGFN RPQMIP--GY NN-----PFQ QQQQPPL--- 634
moss WDR33 -------MQG GPQGYMARSP GIGVV-PGPY MTMPG----- --QLVP--GP PP----LPYQ GGAPPNMMDI 641
rice WDR33 --------QG PPSNM----P PMGGF-PNGM GNIQGGSSAS GMQNFPMGGM YN----RP-- QGKMPPQAQA 491
HsWDR33 MLGPPQELRG PPGSQSQQGP PQGSLGPPPQ GGMQGPPGPQ GQQN-PARGP HPSQGPIPFQ QQKTPLLGDG 913
Pf3D7 WDR33 ---------- ---KVVDKKK ---------- -KKKVKKKIK ---------- --K------- ---------- 468
TgME49 WDR33 ---------- GGGEESDEEE DWGGWMESGR GKPRGLPRIR TRWLVGRHGP AGR------- ---------- 565
Sn SN3 WDR33 SHVDAWVVCG SPTKKRQKN- -----LIPYN GKLKGRLDIT RKW------- ---------- ---------- 701
Conservation
1,060 1,080 1,100 1,120
Arabidopsis WDR33 ---------- ---------- ---------- ----P-PGPP PNNNQQHQ*- ---------- ---------- 648
moss WDR33 RAHGMN---- --VSMGMNVG MGMTMNMG-V PHSHP-SGPP PG----HG*- ---------- ---------- 678
rice WDR33 QMTSIP---- --GLSSYQPG MG---NVG-L PPPPP-QHPP PRGSAP-Q*- ---------- ---------- 528
HsWDR33 PRAPFNQEGQ STGPPPLIPG LGQQGAQGRI PPLNPGQGPG PNKVSEEEPR RGMRAVLPPE EGMVFLVLKT 983
Pf3D7 WDR33 ---------- ---------- ---------- ---------- ---------- ---------- ---------- 468
TgME49 WDR33 ---------- ---------- ---------- ---------- ---------- ---------- ---------- 565
Sn SN3 WDR33 ---------- ---------- ---------- ---------- ---------- ---------- ---------- 701
Conservation
1,140 1,160 1,180
Arabidopsis WDR33 ---------- ---------- ---------- ---------- ---------- ---------- ---------- 648
moss WDR33 ---------- ---------- ---------- ---------- ---------- ---------- ---------- 678
rice WDR33 ---------- ---------- ---------- ---------- ---------- ---------- ---------- 528
HsWDR33 LVQRRILMLL RKRPEDEISE VEVGVPHEEE GRVYFPLLTS SLALKEGGSQ IPGMETESLG QVMNIFVILP 1053
Pf3D7 WDR33 ---------- ---------- ---------- ---------- ---------- ---------- ---------- 468
TgME49 WDR33 ---------- ---------- ---------- ---------- ---------- ---------- ---------- 565
Sn SN3 WDR33 ---------- ---------- ---------- ---------- ---------- ---------- ---------- 701
Conservation
1,200 1,220 1,240 1,260
Arabidopsis WDR33 ---------- ---------- ---------- ---------- ---------- ---------- ---------- 648
moss WDR33 ---------- ---------- ---------- ---------- ---------- ---------- ---------- 678
rice WDR33 ---------- ---------- ---------- ---------- ---------- ---------- ---------- 528
HsWDR33 ALIIPLTTVI PQPAENAPLL SKAWTWHPYL PESAPGMMAQ ALLSTERWRP QEALLKTEEA KAEGAQDLLR 1123
Pf3D7 WDR33 ---------- ---------- ---------- ---------- ---------- ---------- ---------- 468
TgME49 WDR33 ---------- ---------- ---------- ---------- ---------- ---------- ---------- 565
Sn SN3 WDR33 ---------- ---------- ---------- --EAHEDLVL PMDTRSQKII VVPAYHEYLV KMRISRCRVR 739
Conservation
Arabidopsis WDR33 ---------- 648
moss WDR33 ---------- 678
rice WDR33 ---------- 528
HsWDR33 ECPNLGVPAP 1133
Pf3D7 WDR33 ---------- 468
TgME49 WDR33 ---------- 565
Sn SN3 WDR33 LIERTSILII 749
Conservation

D

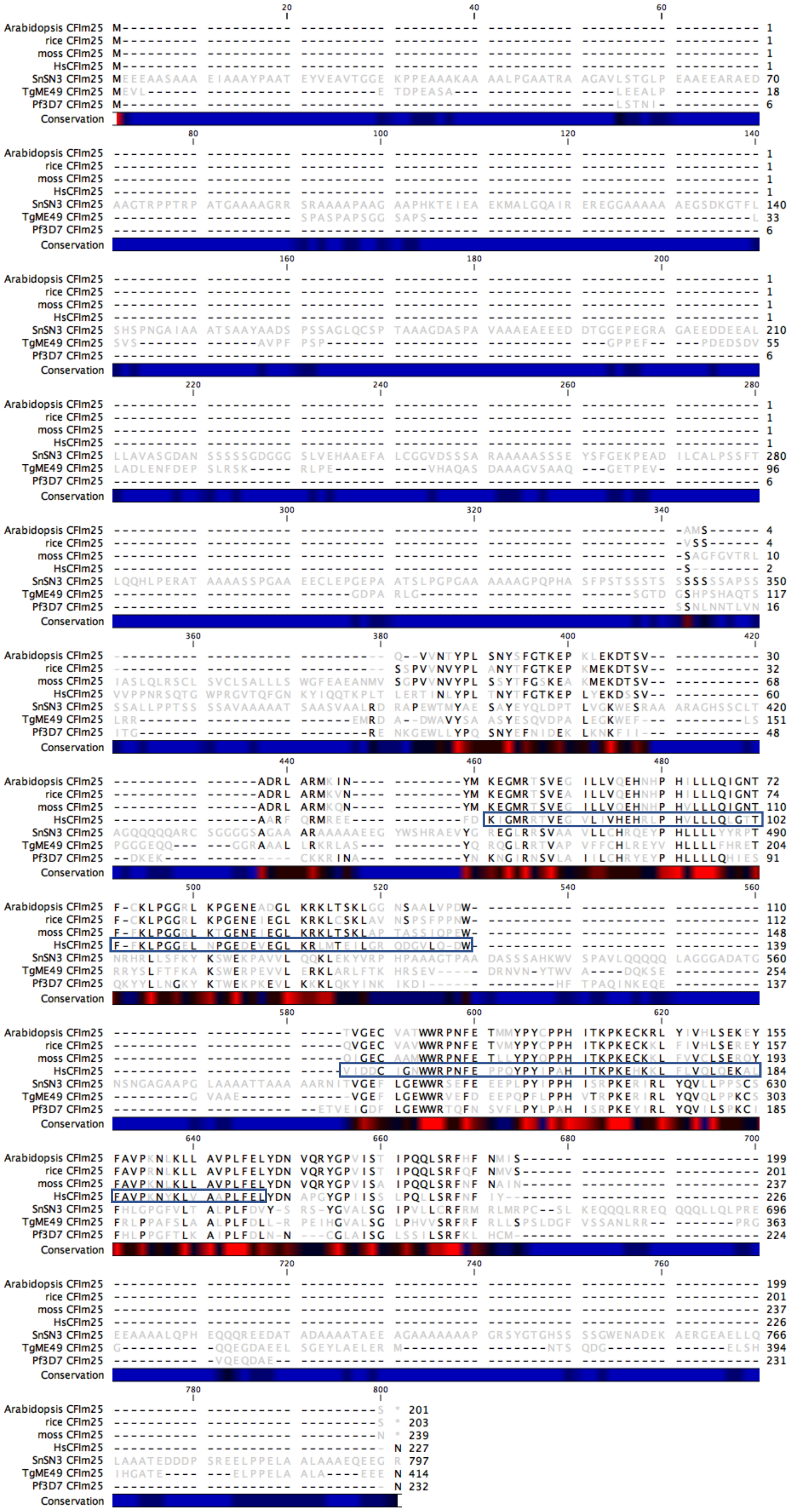
Arabidopsis CFIm25
rice CFIm25
moss CFIm25
HsCFIm25
SnSN3 CFIm25
TgME49 CFIm25
Pf3D7 CFIm25
Conservation

E

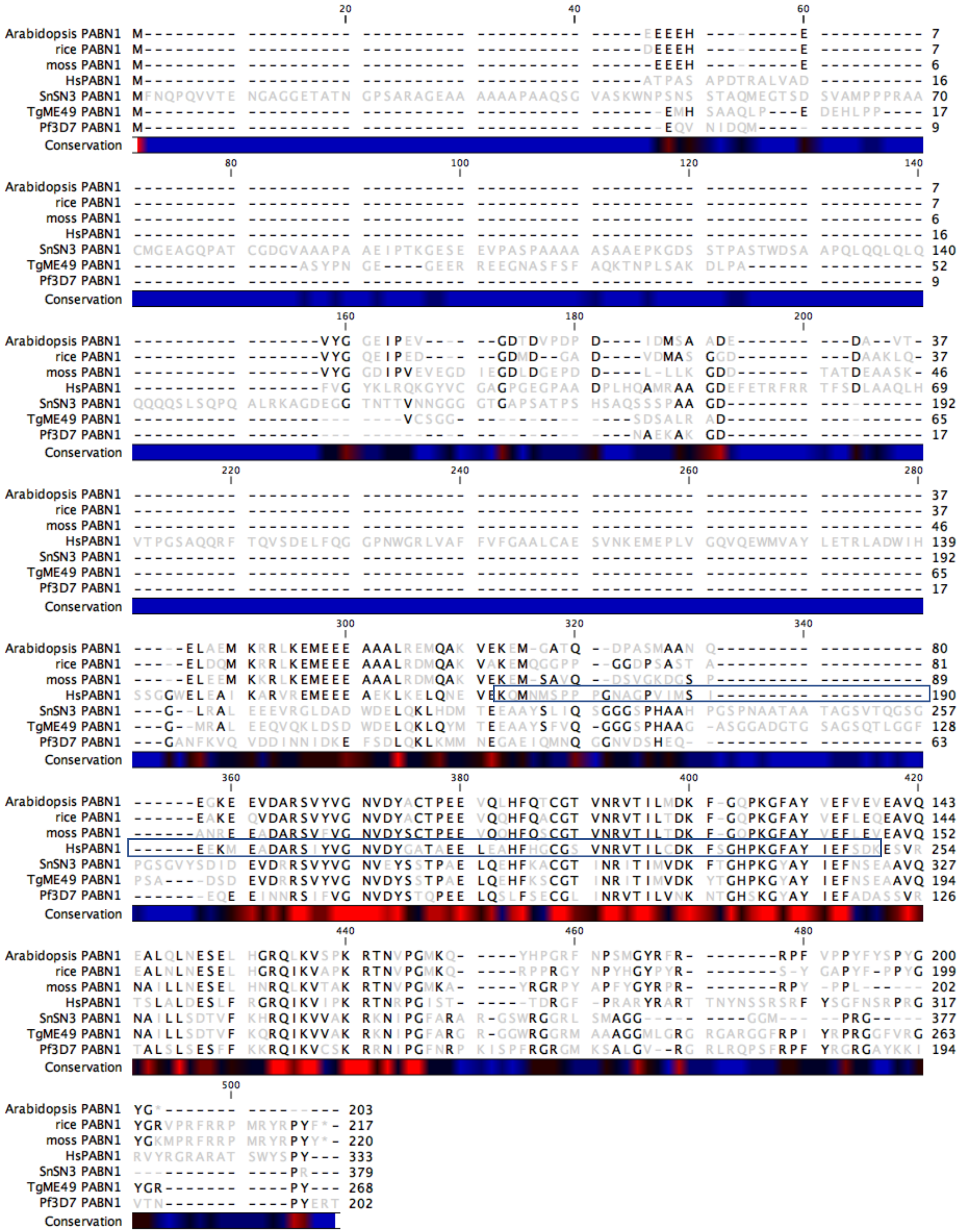
Arabidopsis PABN1
rice PABN1
moss PABN1
HsPABN1
SnSN3 PABN1
TgME49 PABN1
Pf3D7 PABN1
Conservation

F

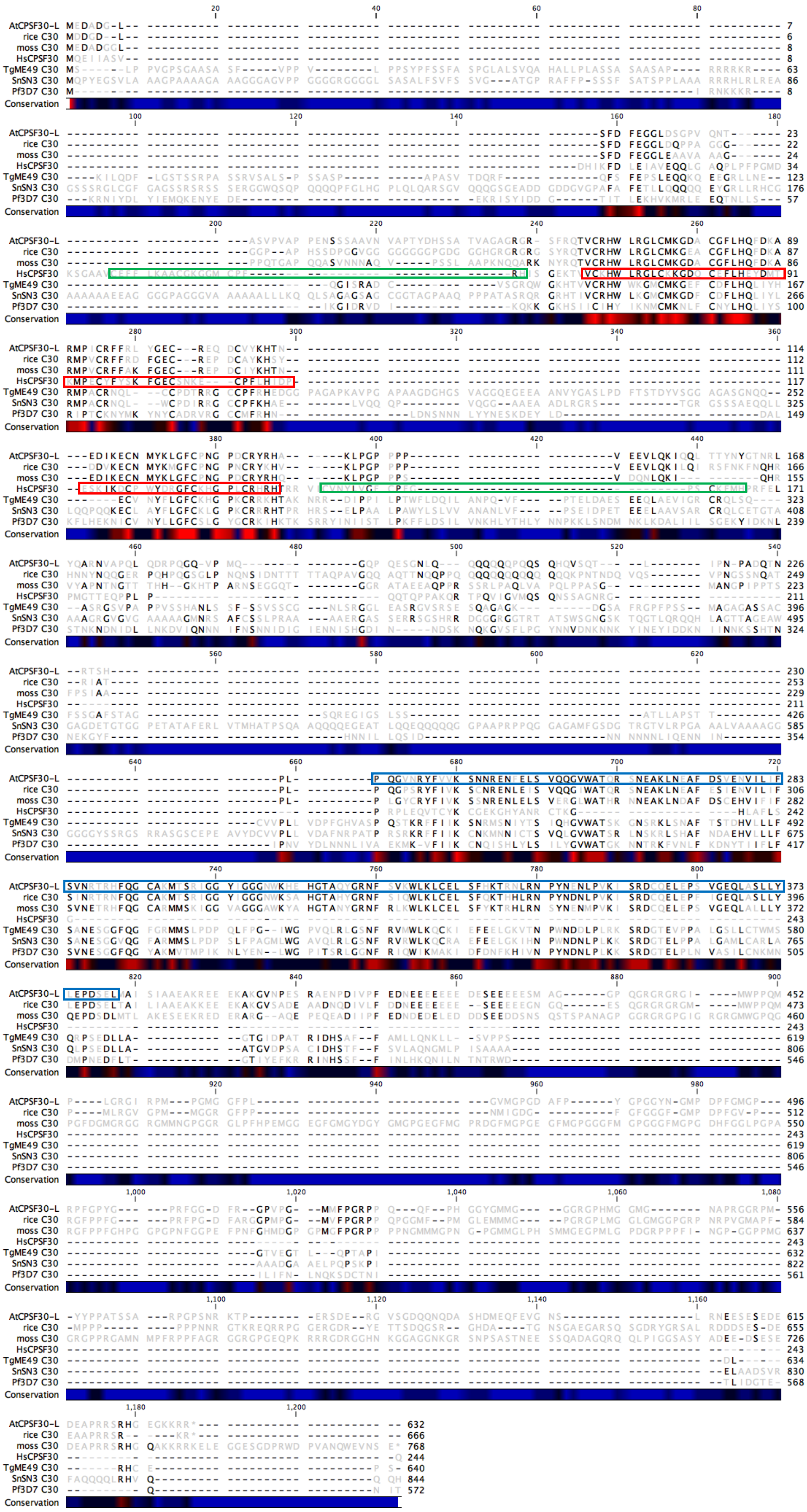
AtCPSF30-L
rice C30
moss C30
HsCPSF30
TgME49 C30
SnSN3 C30
Pf3D7 C30
Conservation

G

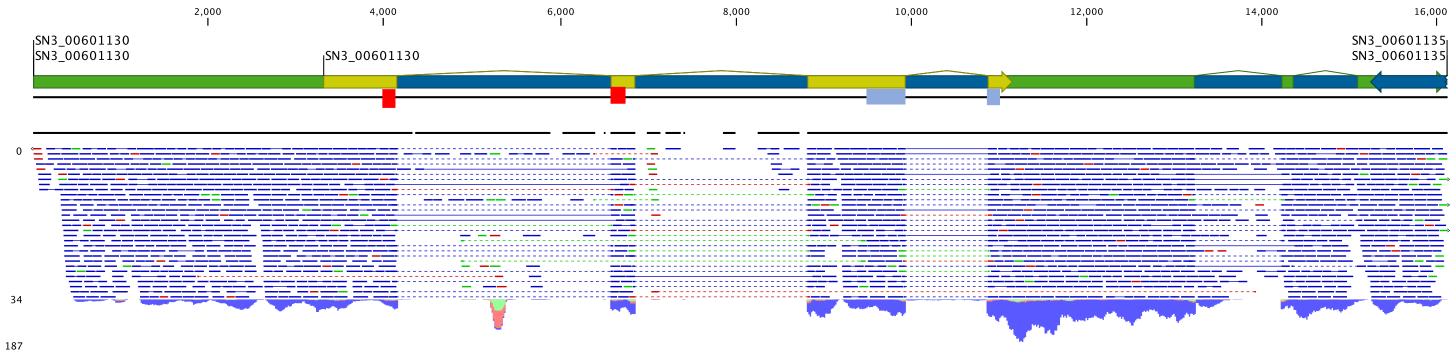
2,000
4,000
6,000
8,000
10,000
12,000
14,000
16,000
SN3_00601130
SN3_00601130
SN3_00601130
SN3_00601135
SN3_00601135
0
34
187
