# Supplementary figures and images for: Characterization of mRNA polyadenylation in the apicomplexa
Source: PLoS One. 2018 Aug 30;13(8):e0203317. doi: 10.1371/journal.pone.0203317 (PMC6117058; doi:10.1371/journal.pone.0203317)

A

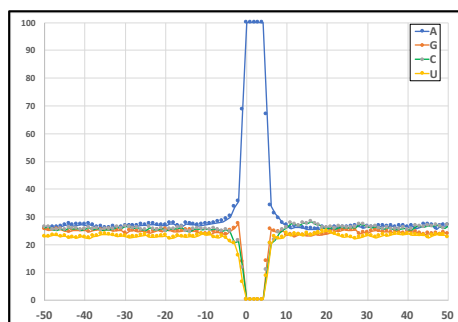

*S. neurona*

B

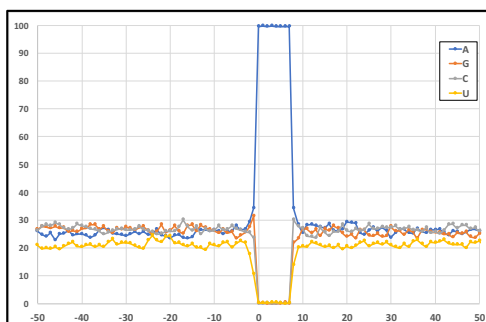

*N. caninum*

C

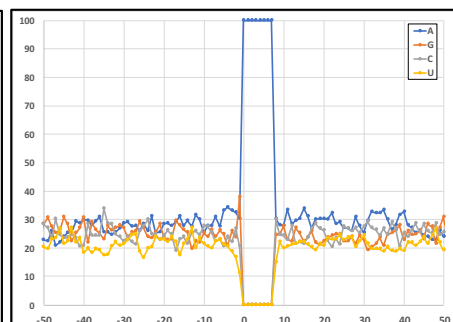

*T. gondii*

Supplement: S2 Fig — Nucleotide composition surrounding tracts of six or more A’s situated within 3’-untranslated regions were identified and the adjacent nucleotide compositions surrounding these plotted in similar fashion. Data points represent the relative fractional composition of each nucleotide at each position extending from 50 nts upstream to 50 nts downstream of the experimentally-determined or mock sites. The organism under study is indicated beneath each plot. (PDF) [file pone.0203317.s002.pdf]

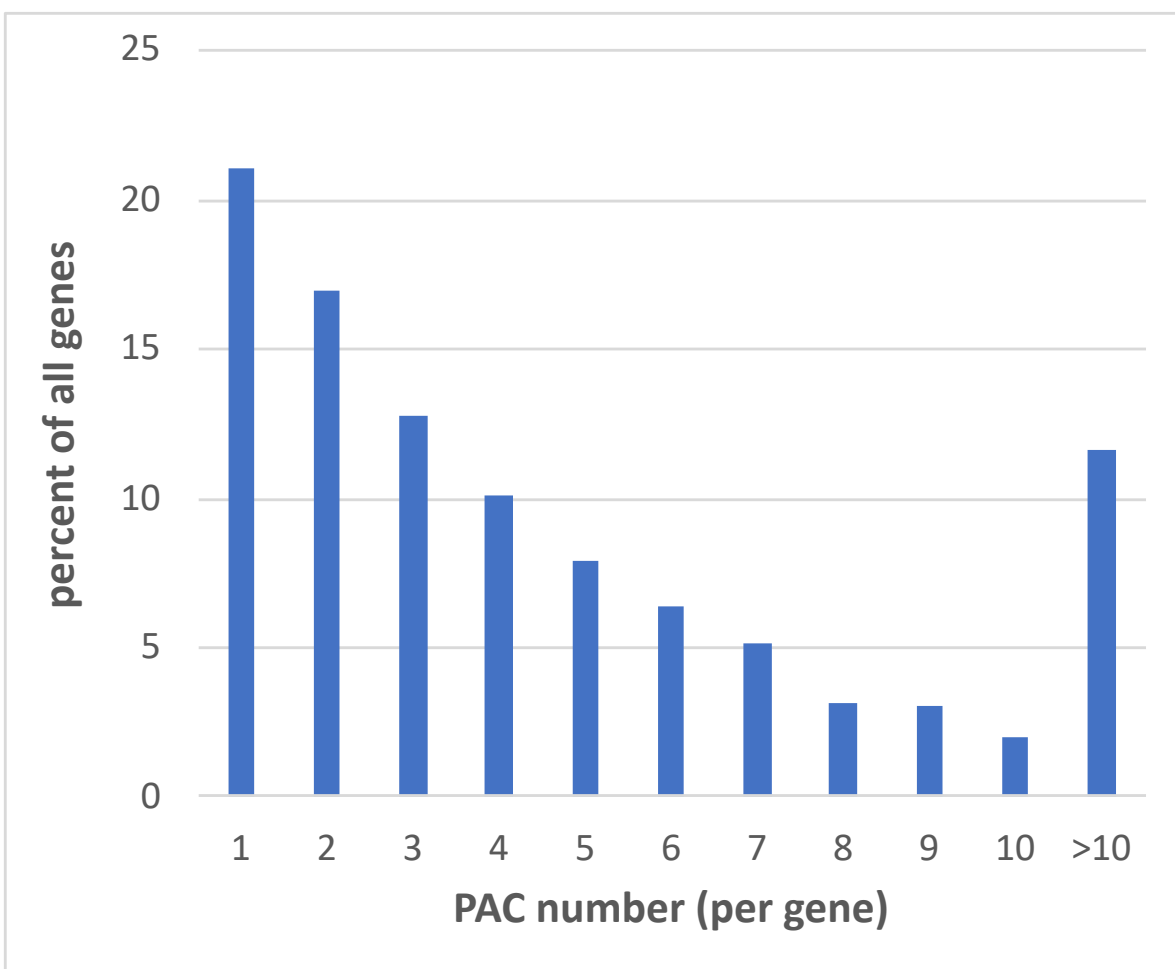

Supplement: S3 Fig — The numbers of PACs per gene were calculated using the data in S4 File and plotted as shown. (PDF) [file pone.0203317.s003.pdf]
