# Supplementary material for: Characterization of mRNA polyadenylation in the apicomplexa
Source: PLoS One. 2018 Aug 30;13(8):e0203317. doi: 10.1371/journal.pone.0203317 (PMC6117058; doi:10.1371/journal.pone.0203317)
Supplement: S1 Table — (DOCX) [file pone.0203317.s009.docx]

Supplemental Table 1. Primers used to prepare PAT-Seq libraries.

| Primer designation | Sequence (5’ -> 3’) | use |
| --- | --- | --- |
| RT-PE3 series | ACACTCTTTCCCTACACGACGCTCTTCCGATCTNNxxxTTTTTTTTTTTTTTTTTTVN | Reverse transcription primers |
| PE-PCR1 | AATGATACGGCGACCACCGAGATCTACACTCTTTCCCTAC  ACGACGCTCTTCCGATCT | Library amplification |
| PE-PCR2 | CAAGCAGAAGACGGCATACGAGATCGGTCTCGGCATTCCT  GCTGAACCGCTCTTCCGATCT | Library amplification |
| SMART7.5 | CGGTCTCGGCATTCCTGCTGAACCGCTCTTCCGATCTGG+G | Strand switching |

Key:

N – random base

xxx – three base bar code used for PAT-Seq libraries

+G – base in a locked nucleic acid conformation
